# Asymmetric Voltage Attenuation in Dendrites Can Enable Hierarchical Heterosynaptic Plasticity

**DOI:** 10.1523/ENEURO.0014-23.2023

**Published:** 2023-07-14

**Authors:** Toviah Moldwin, Menachem Kalmenson, Idan Segev

**Affiliations:** 1 Edmond and Lily Safra Center for Brain Sciences; 2 Department of Neurobiology, The Hebrew University of Jerusalem, 91904 Jerusalem, Israel

**Keywords:** calcium, dendrites, heterosynaptic plasticity, NMDA, synaptic plasticity, VGCC

## Abstract

Long-term synaptic plasticity is mediated via cytosolic calcium concentrations ([Ca^2+^]). Using a synaptic model that implements calcium-based long-term plasticity via two sources of Ca^2+^ — NMDA receptors and voltage-gated calcium channels (VGCCs) — we show in dendritic cable simulations that the interplay between these two calcium sources can result in a diverse array of heterosynaptic effects. When spatially clustered synaptic input produces a local NMDA spike, the resulting dendritic depolarization can activate VGCCs at nonactivated spines, resulting in heterosynaptic plasticity. NMDA spike activation at a given dendritic location will tend to depolarize dendritic regions that are located distally to the input site more than dendritic sites that are proximal to it. This asymmetry can produce a hierarchical effect in branching dendrites, where an NMDA spike at a proximal branch can induce heterosynaptic plasticity primarily at branches that are distal to it. We also explored how simultaneously activated synaptic clusters located at different dendritic locations synergistically affect the plasticity at the active synapses, as well as the heterosynaptic plasticity of an inactive synapse “sandwiched” between them. We conclude that the inherent electrical asymmetry of dendritic trees enables sophisticated schemes for spatially targeted supervision of heterosynaptic plasticity.

## Significance Statement

Our simulations suggest a novel framework for understanding synaptic plasticity. As opposed to plasticity being controlled only locally at the target synapse (as with frequency-dependent protocols) or globally via a backpropagating action potential (as with spike timing-dependent plasticity, STDP), our results indicate that plasticity can be controlled in a sophisticated hierarchical and branch-dependent manner. Our work makes experimentally verifiable predictions for experimentalists studying plasticity and also provides a basis for further theoretical research about dendritic computation and learning.

## Introduction

The brain is believed to learn and store information via modifying the strengths of the synapses between neurons, a process known as long-term plasticity (Hebb, 1949; Bliss and Lomo, 1973; Bliss and Collingridge, 1993; Whitlock et al., 2006; Nabavi et al., 2014; Humeau and Choquet, 2019). Experimentally, plasticity can be induced via a variety of stimulation protocols (Bliss and Lomo, 1973; Rose and Dunwiddie, 1986; Artola et al., 1990; Bliss and Collingridge, 1993; Bi and Poo, 1998; O’Connor et al., 2005b; Shouval et al., 2010). While some plasticity-inducing protocols such as spike timing-dependent plasticity require postsynaptic depolarization (Bi and Poo, 1998), in many cases it is possible to produce long-term potentiation (LTP) or long-term depression (LTD) via presynaptic stimulation alone (e.g., using high-frequency or low-frequency stimulation, respectively; Artola et al., 1990; O’Connor et al., 2005a). Some have argued that presynaptic inputs (without postsynaptic spiking activity) are the primary driver of plasticity in the hippocampus (White et al., 1988; Lisman and Spruston, 2005; Hardie and Spruston, 2009) as well as in some cases in the cortex (Kumar et al., 2021).

**Figure 1.**
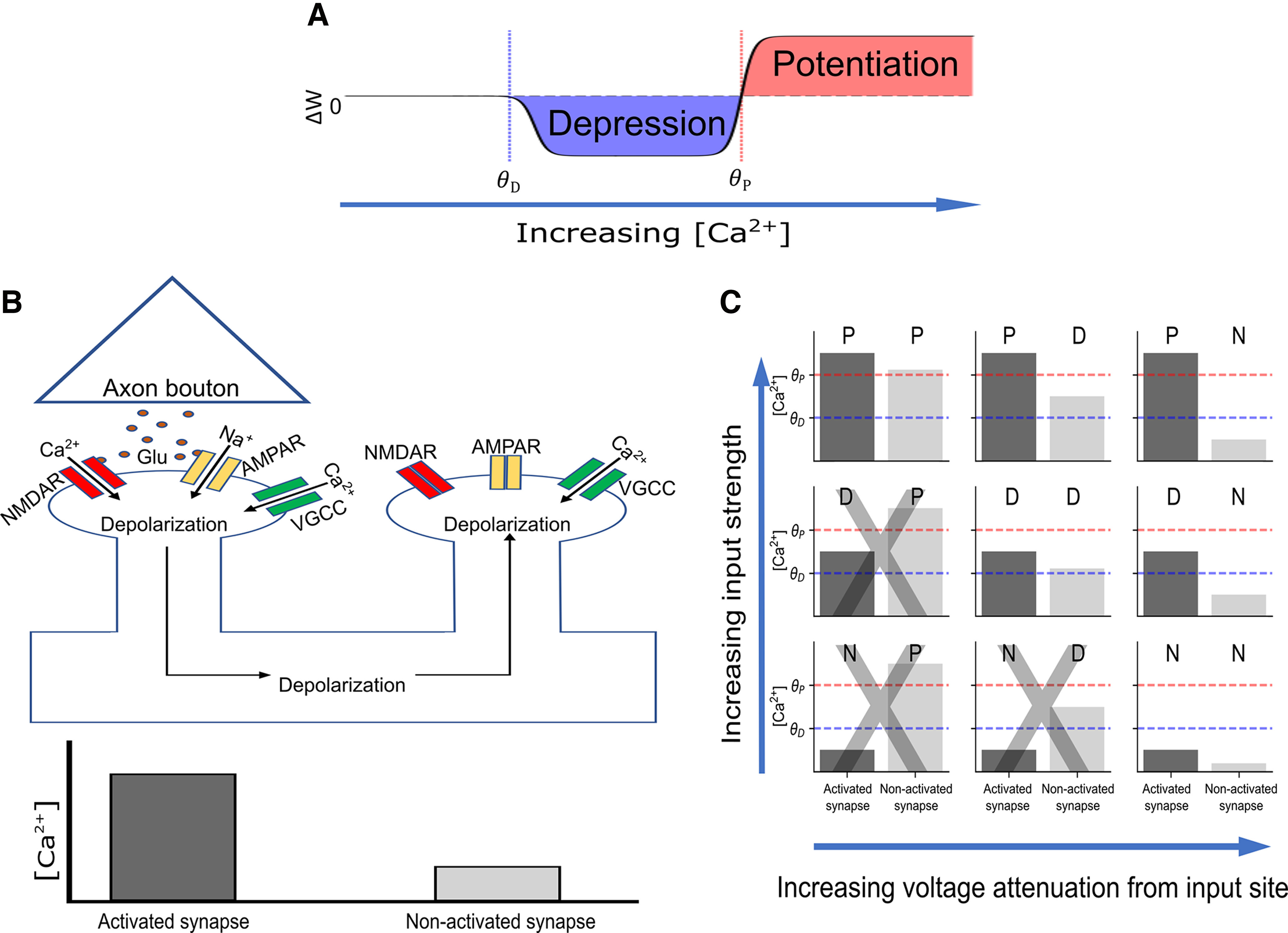
Induction of homosynaptic and heterosynaptic plasticity with NMDA receptors and VGCCs. ***A***, Calcium control hypothesis. The synapse is weakened when the spine [Ca^2+^] crosses the depression threshold $\theta_{D}$ and strengthened when the [Ca^2+^] crosses the potentiation threshold $\theta_{P}$. ***B***, VGCC hypothesis for heterosynaptic plasticity. A presynaptic neuron spikes, releasing glutamate from its axonal bouton which binds to the AMPA and NMDA receptors of the (homosynaptic) postsynaptic spine, causing calcium influx through the NMDA channel and depolarization of the spine. The depolarization also opens the VGCCs in the activated spine, causing additional calcium influx. The depolarization spreads and depolarizes other (heterosynaptic) spines that had not been activated, opening VGCCs in these spines. However, the overall [Ca^2+^] in the nonactivated spines is smaller, as NMDA receptors were not activated. ***C***, Schematic diagram of how input strength (e.g., cluster size) and spatial voltage attenuation can affect homosynaptic and heterosynaptic plasticity. N, No change; P, Potentiation; D, Depression. Panels with a gray “X” indicate scenarios that violate the assumption that activated spines have at least as much [Ca^2+^] as nonactivated spines.

Over the past decades, since first proposed by Lisman (1989), evidence has mounted for a calcium-based theory of plasticity, known as the calcium control hypothesis (Lisman, 1989; Mulkey and Malenka, 1992; Cummings et al., 1996; Yang et al., 1999; Cho et al., 2001; Shouval et al., 2002). In this framework, synapses change their strength depending on the cytosolic calcium concentration ([Ca^2+^]) at the postsynaptic dendritic spine. If the [Ca^2+^] is low, no change occurs. If the [Ca^2+^] rises above a critical threshold for depression ${\text{(θ}}_{\text{D}})$, LTD occurs and the synaptic strength is decreased. If the [Ca^2+^] is above the critical threshold for potentiation $\left({\text{θ}}_{\text{P}}\right)$, LTP occurs and the synaptic strength is increased (Fig. 1*A*). [It is usually assumed that ${\text{θ}}_{\text{P}}>{\text{θ}}_{\text{D}}$ for cortical and hippocampal neurons (Lisman, 1989; Artola et al., 1990), but the reverse may be true for cerebellar Purkinje cells (Coesmans et al., 2004; Piochon et al., 2016)]. It is believed that calcium promotes LTP via pathways involving protein kinases such as calcium/calmodulin-dependent protein kinase II (CaMKII; Lisman, 1989; Malenka et al., 1989; Malinow et al., 1989; Neveu and Zucker, 1996), while promoting LTD via phosphatases such as calcineurin (Lisman, 1989; Mulkey et al., 1993, 1994).

There are several sources of plasticity-inducing calcium at synapses. Two of the most prominent sources are the ligand-gated and voltage-gated NMDA receptor and the voltage-gated calcium channel (VGCC, also known as the voltage-dependent calcium channel, or VDCC). Experimentally-induced plasticity is disrupted or prevented when NMDA receptors or VGCCs are blocked, indicating that the calcium current through these sources is essential for long-term plasticity (Dudek and Bear, 1992; Bi and Poo, 1998; Golding et al., 2002; Fino et al., 2010; Shindou et al., 2011). We note that internal calcium stores can also contribute to long-term plasticity (Nishiyama et al., 2000; Rose and Konnerth, 2001; Royer and Paré, 2003; Jo et al., 2008; Camiré and Topolnik, 2014; Evans and Blackwell, 2015; O’Hare et al., 2022; see Discussion).

One of the original motivations for the calcium control hypothesis (Lisman, 1989, 2001) was the phenomenon of heterosynaptic plasticity: sometimes, when a target synapse is subjected to a plasticity protocol, other nonactivated synapses are affected as well (for review, see Chistiakova et al., 2014; Chater and Goda, 2021). For example, when LTP is induced at a target synapse, other synapses in the neuron can be depressed (Lynch et al., 1977). A calcium-based model can explain this phenomenon if the potentiating protocol produced a large [Ca^2+^] influx (above $\theta_{P})$ in the target spine, and a smaller [Ca^2+^] (above $\theta_{D}$ but below $\theta_{P}$) in nontarget synapses. Lisman (2001) proposed that this might happen in the following manner: when a target synapse is activated, such as by stimulating its presynaptic axons, NMDA receptors in the target spine are activated by the presynaptic glutamate, producing a calcium influx sufficient to potentiate the synapse. In addition to increasing the [Ca^2+^] in the target spine locally, the excitatory current also depolarizes the dendrite. If the depolarization is sufficient to activate VGCCs in other spines, those spines will also experience an influx of calcium, but smaller than that of the target spine, where calcium can accumulate from both NMDA receptors and VGCCs. If the [Ca^2+^] produced by the VGCCs is above $\theta_{D}$ but below $\theta_{P}$, the nontarget synapses (where NMDA receptors are not activated) will depress (Fig. 1*C*).

Heterosynaptic plasticity has also been shown to be spatially sensitive, with different plastic effects being observed at nontarget synapses depending on where they are located relative to the target synapse. Some studies show heterosynaptic plasticity within short distances (∼10 μm) from the target synapse (Royer and Paré, 2003; Chater and Goda, 2021; Tong et al., 2021), whereas other studies show heterosynaptic effects at up to 70 μm away from the activated synapses (Engert and Bonhoeffer, 1997) or even effects that spread from the basal to the apical tree in hippocampal pyramidal neurons (Lynch et al., 1977). While the short-range effects can be potentially explained by molecular diffusion (Chater and Goda, 2021), it is unclear what the underlying principles are that determine the spatial spread of heterosynaptic plasticity over long distances, or what the functional significance of such heterosynaptic changes might be. One experimental finding demonstrated that simultaneous activation of multiple nearby synapses on a dendritic branch can induce branch-level NMDA-dependent and VGCC-dependent calcium signals (Losonczy and Magee, 2006), pointing to the possibility of branch-level plastic changes.

Another issue that arises under the calcium control hypothesis pertains to how simultaneous synaptic input at different regions of the dendrite affects plasticity. It is known that NMDA synapses can interact synergistically such that when multiple nearby synapses are activated simultaneously, the observed somatic EPSP is larger than the linear sum of individual EPSPs, because of the voltage dependence of the NMDA receptor (Polsky et al., 2004). However, how simultaneous synaptic activity at different locations on the dendrite affect plastic changes at both activated and nonactivated synapses was not systematically explored.

Recently, a model synapse was developed as part of the Blue Brain Project (Chindemi et al., 2022) which incorporates NMDA receptors, VGCCs, and calcium-dependent long-term plasticity dynamics. This synapse model (with some modifications described below in Materials and Methods) enables us to explore the Lisman (2001) hypothesis about the calcium basis of heterosynaptic plasticity in a dendritic cable model, which provides insight into the spatial properties of heterosynaptic plasticity.

## Materials and Methods

Simulations were done using NEURON with a Python wrapper (Hines and Carnevale, 1997; Hines et al., 2009). Code was written using Python 3.7.6 and NEURON version 7.7.2. Figures were made with the Matplotlib package and inkscape.

Model parameters for ball-and-stick and branched dendritic model can be found in Table 1. The dendrite models were largely based on the layer 5 pyramidal cell model of Hay et al. (2011) except where described otherwise in Table 1. The dendritic axial resistance and diameter were chosen so as to fit with empirically described results (Yuste and Denk, 1995) and to ensure that a robust NMDA spike could be obtained with activation of ∼20 local excitatory synapses (Eyal et al., 2018). The ball-and-stick model had a dendrite of 200 μm, composed of 50 electrical segments (cylinders).

**Table 1:** Model parameters for ball-and-stick and branched dendritic models

Model parameters	Property	Value	Reference
	*R_a_*	Dendrite: 150 Ωcm	
	*C_m_*	Soma: 1 μF/cm^2^	Hay et al. (2011)
		Dendrite: = 1* μF/cm^2^	Hay et al. (2011)
	*E*_pas_	−77 mV	Hay et al. (2011)
	*R_m_*	Soma: 30 KΩcm^2^	Hay et al. (2011)
		Dendrite*: = 44^*^ KΩcm^2^	Hay et al. (2011)
	*g*_max_	AMPAR (INITIAL): 1.5 nS	Behabadi et al. (2012)
		AMPAR (UP): 2 nS	
		AMPAR (DOWN): 1 nS	
		VGCC: ∼0.2 nS**	Fisher et al. (1990); Snutch et al. (2013); Tsien et al. (1988)
		NMDAR: 1.31 nS	Eyal et al. (2018)
Morphologic parameters	Diameter	Dendrite: 0.75 μm	Araya et al. (2006)
		Soma: 718 μm	See Materials and Methods and Extended Data Fig. 2-1
	Length	Dendrite (ball and stick): 200 μm	
		(Branched model): 50 μm/branch	
		Soma: 23 μm	Hay et al. (2011)
Spine parameters	*R_a_*	150 Ωcm	
	*R_m_*	10.7 KΩcm^2^	Hay et al. (2011) (no spine comp.)
	Diameter	Head: 0.4 μm	Konur et al. (2003)
		Neck: 0.07 μm	Arellano et al. (2007) (fit to ensure R_neck_ of 226.6 MΩ)
	Length	Neck: 0.66 μm	Arellano et al. (2007)
	*R*_neck_	Neck: 226.6 MΩ	Cornejo et al. (2021)

* *R_m_* used in the simulation was divided by 2 and C_m_ multiplied by 2 to compensate for surface area of unmodeled spines while maintaining the membrane time constant (Eyal et al., 2018).

** Overall conductance in the spine (gca_bar_abs_VDCC), based on the assumption of 20 pS unitary conductance per calcium channel and 20 channels per μm^2^ (Sabatini and Svoboda 2000).

**Figure 2.**
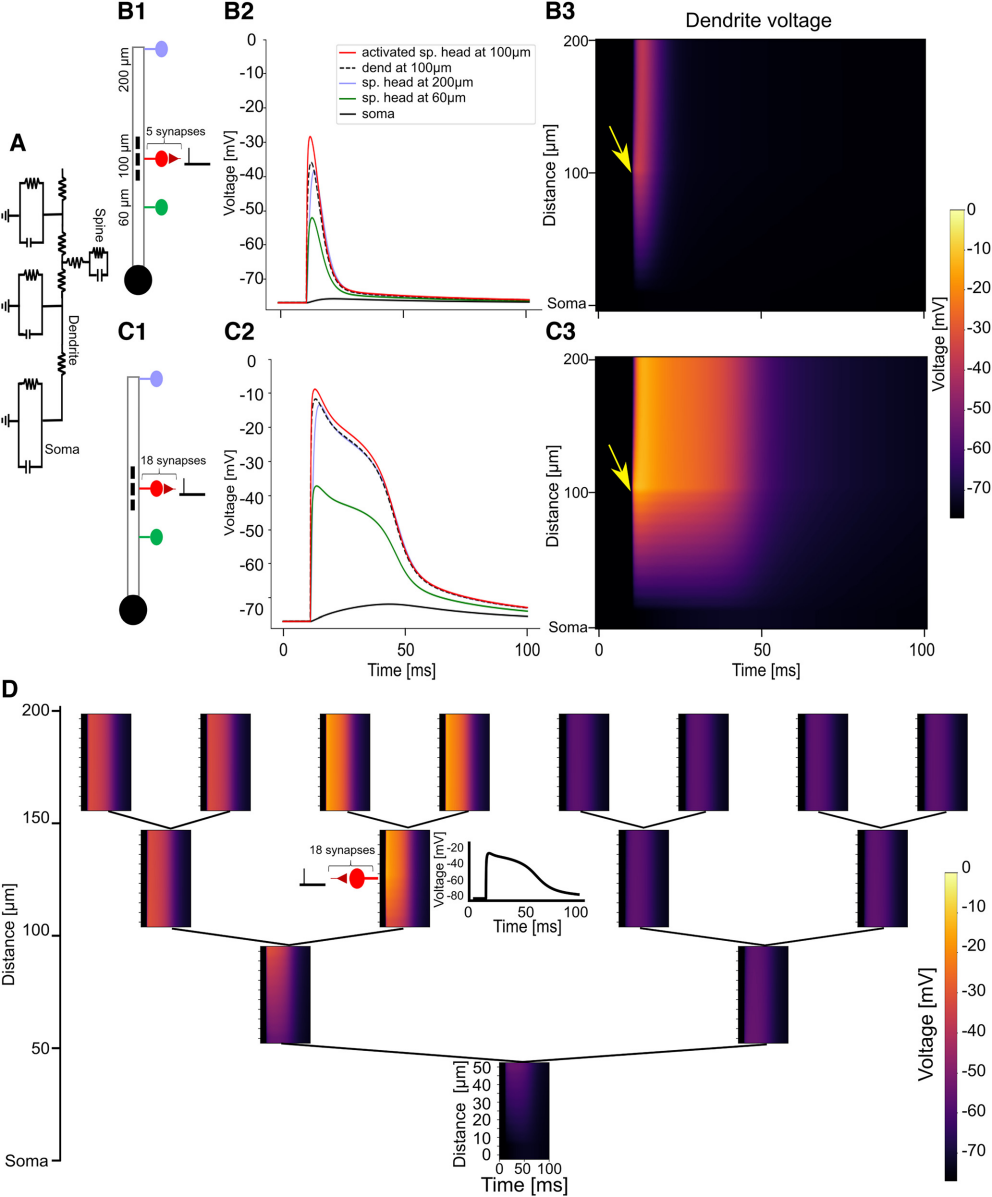
Asymmetric attenuation of EPSPs and NMDA spikes in dendritic cable models. ***A***, Circuit diagram of a ball-and-stick dendritic model with spines. See Extended Data Figure 2-1 for transfer resistance comparison with pyramidal neuron model branch. ***B1***, Experiment schematic. A cluster of 5 spine synapses located at 100 μm from the soma are simultaneously activated. Voltage is recorded from one of the activated spine heads (red spine), its base (black dashed line), a nonactivated spine at 200 μm from the soma (blue spine), and a nonactivated spine at 60 μm from the soma (green spine) and the soma (black solid circle). ***B2***, Voltage traces from recording sites depicted in ***B1***. Voltage is largest at the activated spine head (red solid line); it attenuates somewhat to the spine base (dashed black line). Very little attenuation occurs from the activated spine base to the distal spine head (blue), but significant attenuation is observed toward the proximal spines (green line) and the soma (black line). ***B3***, Voltage recordings along the dendrite during the experiment depicted in ***B1***. Color depicts dendritic voltage as a function of time (horizontal axis) and distance from soma (vertical axis). Arrow indicates time and location of the activated synaptic cluster. ***C1–C3***, Same as ***B1–B3*** except a cluster of 18 synapses are simultaneously activated to generate an NMDA spike. See Extended Data Figure 2-2 for comparison with pyramidal neuron model branch and models with constant and scaled *I*_h_ conductance. ***D***, Dendritic voltage heatmaps in each branch of an order-3 branching dendritic model in response to an NMDA spike initiated via activating a cluster of 18 synapses at the indicated dendritic location. Inset, Voltage trace at the base of the activated spine cluster. See Extended Data Figure 2-3 for voltage responses when activated synapses are less densely clustered. See Extended Data Figure 2-4 for results with asymmetric branching morphology.

The branching dendritic model was an order-3 branching dendritic tree (i.e., three bifurcating branching levels emanating from the zero-order main branch) coupled to a soma compartment (Fig. 2*D*). Each branch section was 50 μm long, resulting in a path length of 200 μm from the soma to the distal tip of each branch of the dendritic tree, consistent with the total dendritic length of the ball-and-stick model. The branched model has the same biophysical parameters as the ball-and-stick model. Each 50-μm-long branch was composed of 10 electrical segments.

To create a ball-and-stick model that replicates the electrical sink effect observed in the soma of a neuron with a full complement of extended dendrites, we expanded the diameter of the soma in the ball-and-stick model so as to have the same input resistance by applying the formula $R_{N}=\frac{R_{m}}{A}$, where $R_{N}$ is the somatic input resistance of the pyramidal cell model in Ω, $R_{m}$ is the membrane resistivity in Ωcm^2^, and A is the area of the compensated soma in cm^2^, resulting in a compensated soma diameter of 718 μm. As in the Hay et al. (2011) model, effective dendritic membrane resistivity $\text{dendritic }R_{m}$ is divided by 2 to compensate for the surface area of (unmodeled) spines, and dendritic membrane capacitance $\text{dendritic }C_{m}$ was doubled to ensure that the membrane time constant τ_m_ does not change.

10.1523/ENEURO.0014-23.2023.f2-1 Figure 2-1 Asymmetric transfer resistances of L5PC and ball-and-stick models. ***A1***, Model of layer 5 pyramidal cell from the study by Hay et al. (2011). Arrow indicates a dendrite modified to have the same morphological and passive electrical parameters as the idealized ball-and-stick model. ***A2***, Pairwise transfer resistances between each dendritic segment, presented as heatmap. L1. location 1, L2, location 2. ***A3***, Transfer resistances from selected dendritic locations (L1) to all other dendritic locations (L2) as a function of distance of L2 from the soma. ***A4***, Same as in ***A3***, except L2 is depicted as a function of distance from L1, demonstrating asymmetric attenuation from each location. Negative numbers indicate L2 is more proximal than L1, and positive numbers indicate L1. ***B1–B4***, Same as in ***A1–A4***, but for the ball-and-stick model used in the article with the soma diameter adjusted to match the somatic input resistance ($R_{N}$) from the Hay L5PC model. Download Figure 2-1, TIF file.

10.1523/ENEURO.0014-23.2023.f2-2 Figure 2-2 Attenuation in dendritic branches with active channels. ***A1***, A cluster of 18 synapses are activated in the center of the modified branch from the study by Hay et al. (2011) model with active channels. ***A2***, Voltage traces from the activation location (100 μm from soma) and other locations on the branch and soma. Compare with Figure 2*C2*. ***A3***, Heatmap of dendritic activation over space and time in the dendritic branch model. Compare with Figure 2*C3*. ***B1***, Same experiment performed on ball-and-stick model with a constant (as a function of distance from soma) *I*_h_ conductance of 2 pS/μm^2^ along the dendrite, as in the basal dendrites of the original model from the study by Hay et al. (2011). ***C***, Same experiment performed in ball-and-stick model with *I*_h_ conductance scaled with distance from soma such that the *I*_h_ conductance at the distal tip is near 400 pS/μm^2^ to imitate the *I*_h_ found in distal apical dendrites of pyramidal neurons 1000 μm from the soma (Kole et al., 2006). Scaling was performed according to the equation $g_{Ih}=-2+428+e^{\frac{x}{0.135*323}}$ pS/μm^2^, where *x* is the distance from the soma. Download Figure 2-2, TIF file

**Figure 3.**
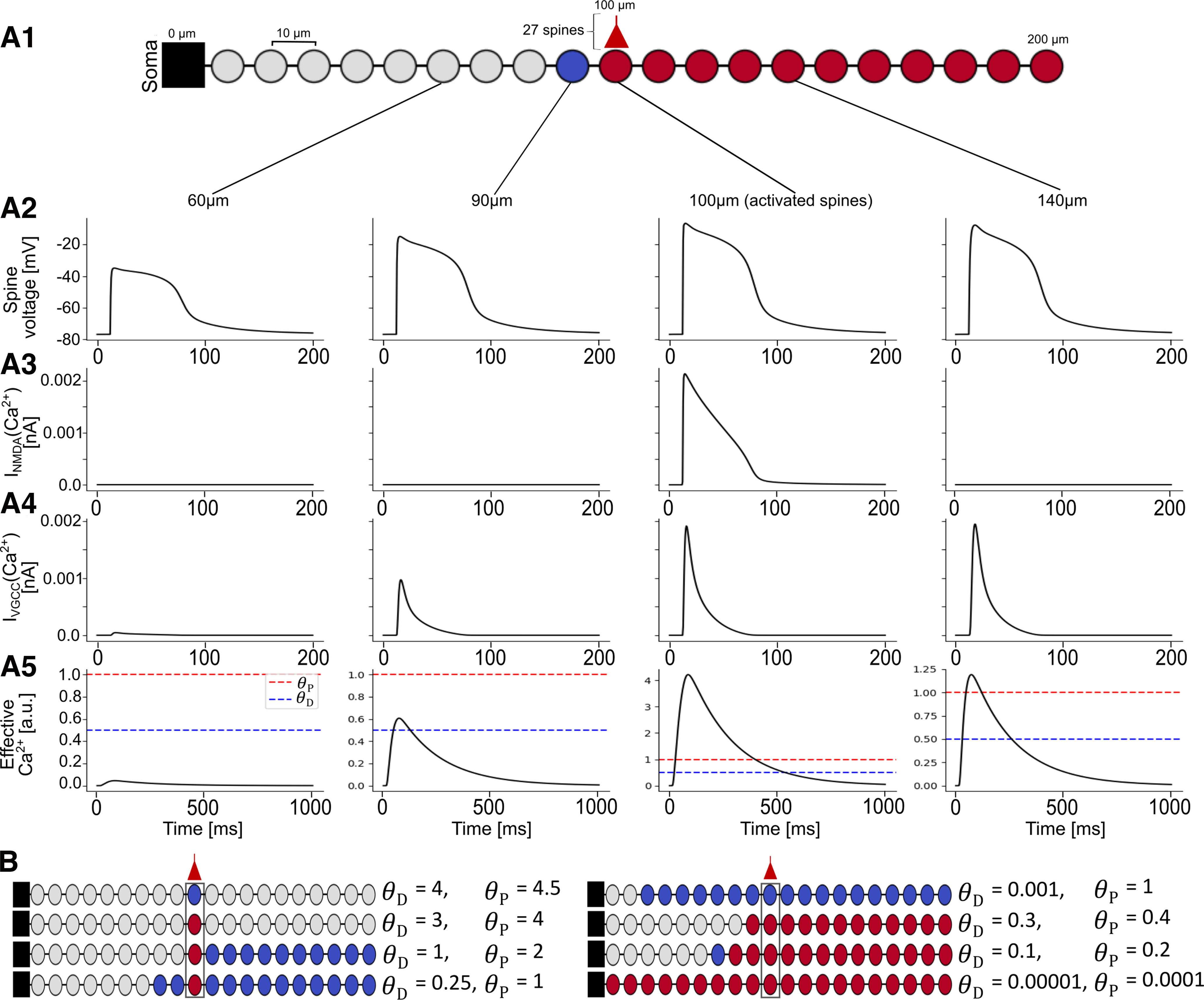
Asymmetric heterosynaptic plasticity induced by VGCCs. ***A1***, Top, A ball-and-stick model dendrite with spines (circles) placed every 10 μm. A cluster of 27 spines (shown as a single circle with an input) is activated at the center of the dendrite (100 μm), generating an NMDA spike that results in homosynaptic and heterosynaptic plasticity. The activated spines and the spines distal to it are potentiated (red), the spine 10 μm proximal to the activated spine is depressed (blue), and the other proximal spines do not change (gray). ***A2***, Spine head voltage traces shown at 60 μm, 90 μm, 100 μm (exemplar activated spine), and 140 μm. The NMDA spike is seen at all spines, but the voltage at the proximal location (60 μm) is substantially attenuated. ***A3***, Ca^2+^ current through the NMDA receptor at the 4 depicted spines. Only the activated spine has NMDA current because NMDA receptors are ligand gated. ***A4***, Ca^2+^ current through the VGCCs at the depicted spines; Ca^2+^ current depends on local voltage (shown in ***A2***). ***A5***, Effective [Ca^2+^] at the depicted spines. At 60 μm, the [Ca^2+^] is below $\theta_{D}$ (blue dashed line), so no change occurs; at 90 μm, the [Ca^2+^] reaches above $\theta_{D}$ but below $\theta_{P}$ (red dashed line), so depression occurs; at 100 and 140 μm, the [Ca^2+^] reaches above $\theta_{P}$, so the synapses are potentiated. ***B***, As in ***A1*** (27 synapses activated at 100 μm) but with different calcium thresholds for plasticity, resulting in different heterosynaptic effects.

10.1523/ENEURO.0014-23.2023.f2-3 Figure 2-3 Effect of clustered versus distributed synaptic distribution. ***A1***, Attenuation in branching dendritic model with synapses clustered at a single dendritic segment, as in Figure 2*D*. ***A2***, Same as in ***A1***, except synapses are uniformly distributed along the branch (18 synapses spread over 50 μm, a density of 0.36 synapses/μm). ***B***, Comparison of voltage traces from the proximal and distal ends (1 and 50 μm, respectively) and midpoint (25 μm) of the activated branch in the clustered and distributed scenarios. Download Figure 2-3, TIF file.

**Figure 4.**
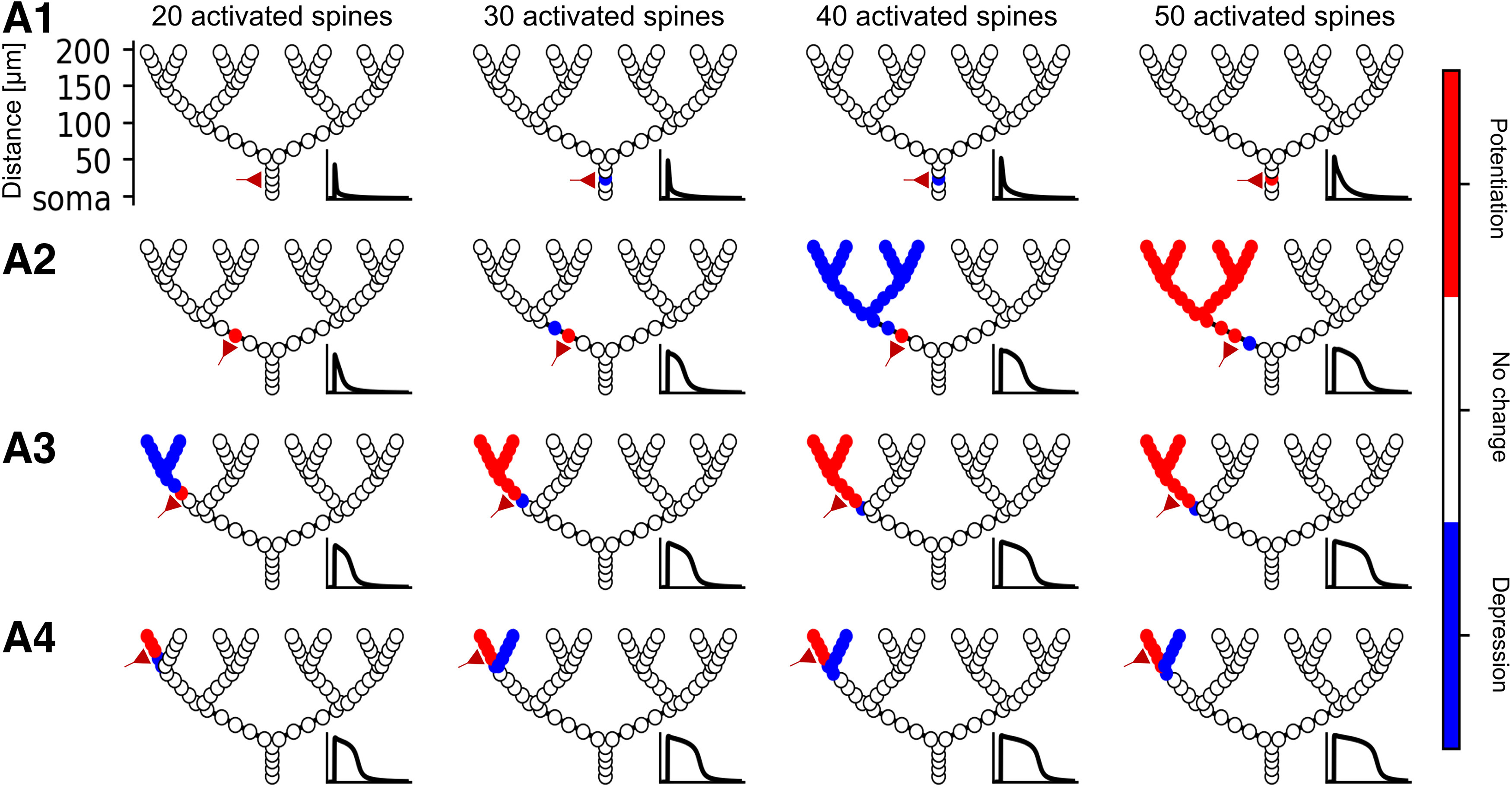
Hierarchical heterosynaptic plasticity in a branching dendritic model. ***A1***, A synaptic cluster of 20 (first column), 30 (second column), 40 (third column), or 50 (fourth column) spines are simultaneously activated at the indicated location (schematic red synapse) on the proximal zero-order branch. Insets, Spine head voltage at the activated sites. Because of the low input resistance at the proximal branch, 20 synapses are insufficient to generate homosynaptic plasticity, while 30 and 40 synapses generate local homosynaptic depression (blue circles), and 50 synapses generate homosynaptic potentiation (red circles). ***A2***, Same as ***A1***, but the activated cluster is now at the first-order branch. Twenty activated synapses create homosynaptic potentiation, 30 or 40 activated synapses create a short-duration NMDA spike and heterosynaptic depression at synapses distal to the activation site, whereas 50 activated synapses create a prolonged NMDA spike and heterosynaptic potentiation at distal sites. ***A3***, Activated cluster at the second-order branch; 20 activated synapses cause a short NMDA spike and distal heterosynaptic depression; ≥30 synapses create a prolonged NMDA spike and heterosynaptic potentiation at distal synapses. ***A4***, Activated cluster at third-order branch. Twenty activated synapses are sufficient to cause distal heterosynaptic potentiation, and ≥30 activated synapses also produce heterosynaptic depression at the sister branch to the input site.

10.1523/ENEURO.0014-23.2023.f2-4 Figure 2-4 Attenuation in asymmetric dendritic morphologies. ***A***, ***B***, Synapses are placed in the indicated location on a dendritic tree with different morphologies: order-1 branching dendrite (***A***); and asymmetric dendritic morphology with additional branching level on the left side with synapse placed on left branch (***B***). ***C***, As in ***B*** but synapse placed on the right branch. ***D***, Order-2 dendritic tree. Download Figure 2-4, TIF file.

To validate that the dendritic attenuation profile of our ball-and-stick model with its compensated soma diameter indeed replicated the attenuation profile one would observe in a real neuron, we selected one of the basal dendritic branches in the L5PC neuron and modified it to have the same length and passive electrical properties as the dendrite in the ball-and-stick model. We computed the point-to-point transfer resistance between each dendritic segment in both the ball-and-stick model and the branch from the L5PC cell model. A comparison of the resultant transfer resistance matrices demonstrated that the two models possessed the same passive electrical properties. Moreover, it was easy to see the pronounced asymmetric attenuation effect in both the L5PC model and the ball-and-stick model from the transfer resistance matrices; the transfer resistances from all points on the dendrite exhibited a rapid drop toward the proximal direction while remaining relatively constant toward the distal direction (Extended Data Fig. 2-1).

For synapses, we modified the Blue Brain Project synapse model with NMDA receptors, VGCCs, and calcium-based long-term plasticity model (Chindemi et al., 2022); the original unmodified synapse file (GluSynapse.mod) can be found at https://zenodo.org/record/6352774. The calcium-based plasticity model itself is based on the work of Graupner and Brunel (2012).

We made the following several modifications to the Blue Brain synapse: (1) we initialized the synapses in a neutral state of 1.5 nS (equivalent to ρ=0.5; i.e., the unstable fixed point in the Graupner–Brunel model), so synapses could be easily depressed or potentiated when the calcium accumulator crossed the plasticity thresholds; (2) we changed the maximum conductance of the NMDA receptor (gmax_NMDA) to 1.31 nS based on the study by Eyal et al. (2018); (3) we increased the unitary conductance of the VGCCs to 20 pS based on previous studies (Tsien et al., 1988; Fisher et al., 1990; Snutch et al., 2013), and assumed a channel density of 20 channels per μm^2^ (Sabatini and Svoboda 2000). (While the kinetics of the VGCC model are based on the R-type VGCC, 20 pS was chosen as an approximate estimate of the typical unitary conductance over all high-voltage activated channels); and (4) we modified the equations for total spine calcium conductance and concentration to account for the fact that we were modeling the spine as a cylindrical neck with a spherical head with the parameters in Table 1. Code for the revised synapse (GluSynapse_TM_MK.mod), and all the models and simulations in this article can be found in the repository linked in the Data availability subsection.

**Figure 5.**
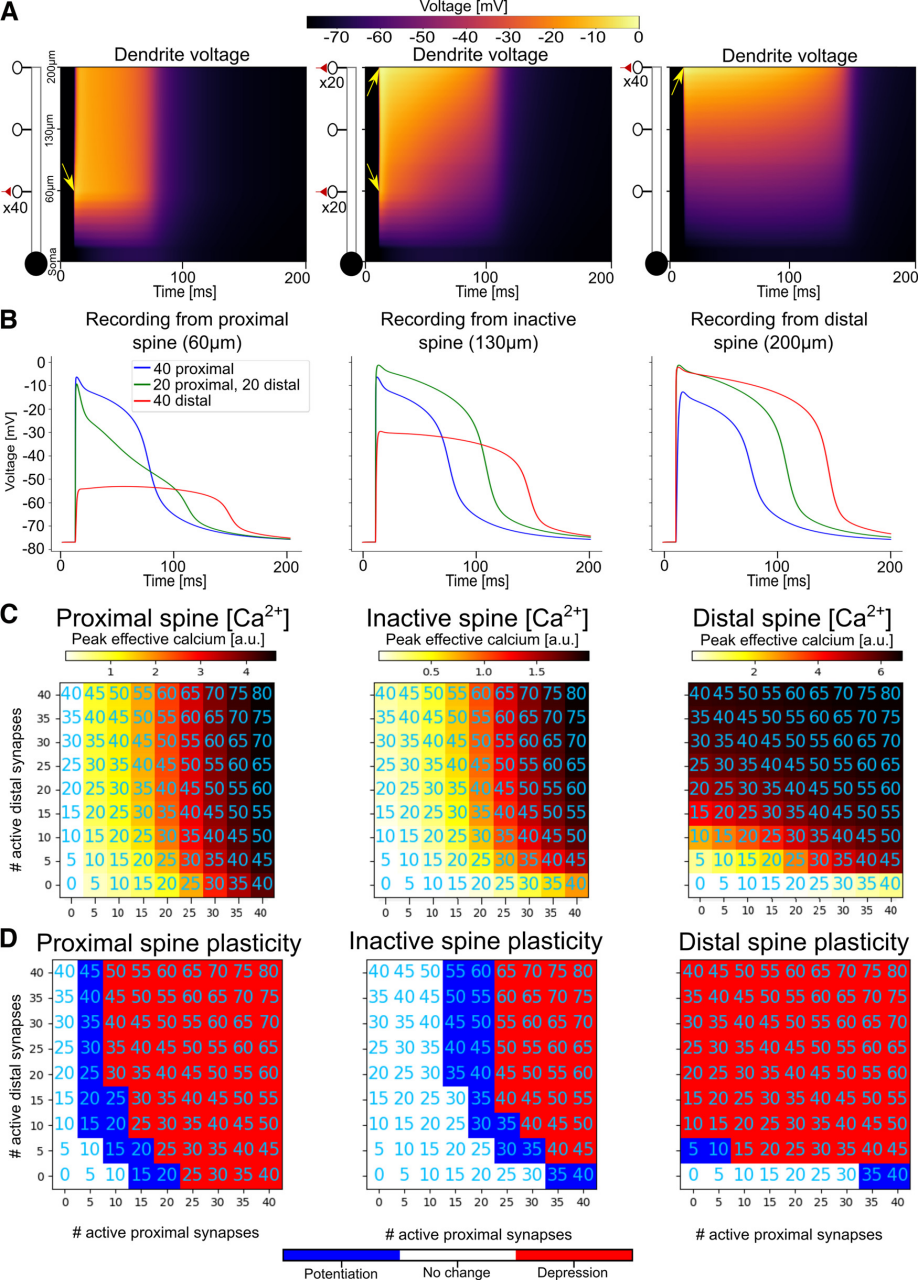
Proximal and distal clusters can create synergistic heterosynaptic effects for “sandwiched” synapses. ***A***, Dendritic voltage over time in a ball-and-stick model (schematic at left) when 40 synapses are placed proximally (left) or distally (right), or are distributed evenly between the proximal and distal locations (center). Arrows show the location and time of the activated synapses. ***B***, Spine head voltage recordings from a proximal activated spine (left), the central nonactivated spine (center), and a distal activated spine (right) for the 3 cases shown in ***A***. ***C***, Peak calcium influx at the proximal (left), central (middle), or distal spine (right) as a heatmap for different numbers of spines placed in the distal and proximal clusters. Annotations indicate total number of activated spines (proximal + distal). ***D***, Plastic effect (red, potentiation; blue, depression; white, no change) resulting from the calcium influx shown in ***C***.

**Figure 6.**
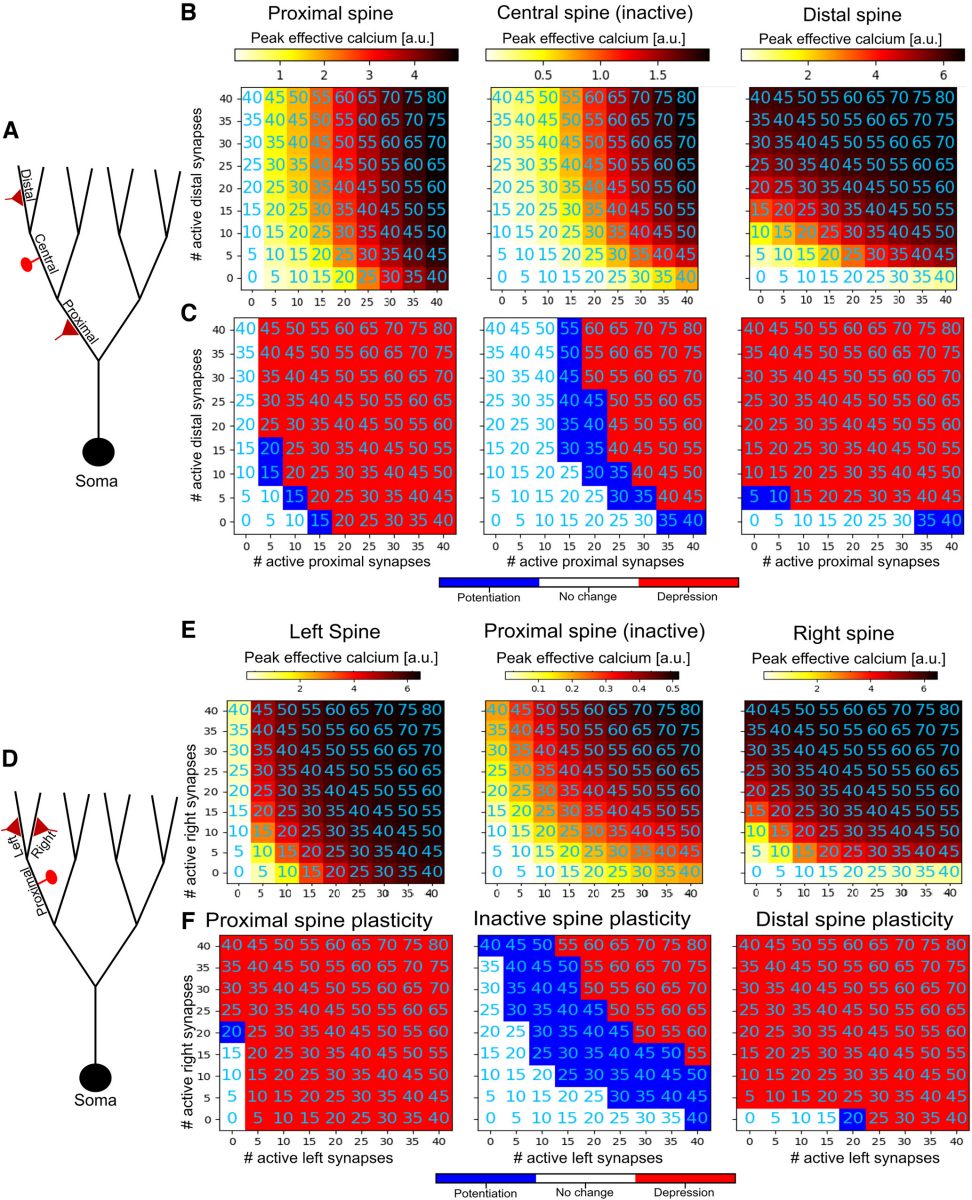
Vertical and horizontal heterosynaptic sandwiching in a branched dendritic model. ***A***, Experiment schematic for vertical sandwiching in a branched model. Clusters of spines are activated at proximal (first-order) and distal (third-order) branches to explore their effect on each other and on a nonactivated spine located at a “central” branch (second order) in between them. ***B***, Peak calcium concentration (in A.U.) at an exemplar spine on the proximal (left), central (middle), or distal (right) branches. ***C***, Plastic effect on each spine as a function of cluster sizes (red, potentiation; blue, depression; white, no change). See Extended Data Figure 6-1 for voltage traces and heatmaps. ***D***, Experiment schematic for horizontal sandwiching in a branched model. Clusters of spines are activated at third-order left and right branches to explore how clusters on sister branches affect each other as well as a nonactivated synapse on their parent (second-order) branch. ***E***, ***F***, Calcium and plasticity for the horizontal sandwiching experiment as in ***C*** and ***D***. See Extended Data Figure 6-2 for voltage traces and heatmaps.

Except where indicated, for Figures 3-6*C*, plasticity thresholds for the [Ca^2+^] were as follows: $\theta_{D}=0.5$ and $\theta_{P}=1$. For Figure 6*D–F* and Extended Data Figure 6-2, plasticity thresholds were $\theta_{D}=0.2$ and $\theta_{P}=0.4$. Plasticity thresholds can vary from cell to cell, so all plasticity results presented in all figures should be taken as qualitative illustrations of possible plastic effects rather than specific quantitative predictions.

### Data availability

Code for this project can be found at https://github.com/mkblitz/Hierarchical-hetero.

## Results

### Possible heterosynaptic effects

We begin by considering the range of possible heterosynaptic effects that may occur according to the hypothesis that homosynaptic plastic effects from presynaptic plasticity induction protocols are induced by calcium influx from both NMDA receptors and VGCCs, whereas heterosynaptic effects are induced only via calcium influx through VGCCs. In this view, a spine activated with presynaptic input will almost inevitably have a higher calcium concentration than nonactivated spines, as both NMDA receptors and VGCCs can enable calcium influx in the activated spine, but only VGCCs can be opened in the nonactivated spine (Fig. 1*B*).

Because calcium thresholds for LTP and LTD can vary from cell to cell (Yang et al., 1999), and can also be changed via metaplastic processes (Abraham and Bear, 1996), we generically map out several possible results that can occur to an activated and nonactivated synapse given a few basic assumptions, as follows: (1) an activated synapse has a higher calcium influx than a nonactivated synapse; (2) plasticity thresholds and voltage-gated channel densities are approximately the same from spine to spine within the same neuron; and (3) that the [Ca^2+^] threshold for potentiation is higher than that of depression (i.e., $\theta_{P}>\theta_{D}$). We also disregard the magnitude of the plastic change and only consider the direction (potentiation or depression), as we assume that after inducing plasticity, synapses eventually drift toward a binary potentiated or depressed state, based on (Graupner and Brunel, 2012). (Some other assumptions also apply; for more details, see Discussion.)

Given these assumptions, the following plastic effects can result (Fig. 1*C*): if the activated synapse is potentiated, nonactivated synapses can also be potentiated (PP), but they can also be depressed (PD), or undergo no change (PN). If the activated synapse is depressed, nonactivated synapses can also be depressed (DD) or undergo no change (DN). If the activated synapse does not change, neither will the nonactivated synapse (NN). Given our assumptions above, the following possibilities are not possible: DP, NP, and ND (Fig. 1*C*). (In the event that $\theta_{D}>\theta_{P}$, as in Purkinje cells, the allowed possibilities are PP, PN, DP, DD, DN, NN, and the disallowed possibilities are PD, NP, and ND; however, the simulations used in this study assume $\theta_{P}>\theta_{D}$ to match known results from the hippocampus and cortex (Mulkey and Malenka, 1992; Cho et al., 2001). We also assume that the input to the activated synapse is not sufficiently large to put it into a ‘postpotentiative neutral zone’ where the calcium concentration is so high that potentiation mechanisms are inactivated (Tigaret et al., 2016).

Intuitively, given values for $\theta_{D}$ and $\theta_{P}$, homosynaptic effects from presynaptic input protocols will vary based on the “strength” of synaptic input (e.g., input frequency or cluster size, as we use here). Strong inputs can potentiate the synapse, medium strength inputs can depress it, and weak inputs will induce no change. As heterosynaptic effects are mediated by dendritic depolarization, heterosynaptic effects will also depend on the input strength to the activated synapse, in addition to other factors that determine the “spillover effect” of the dendritic depolarization of the activated synapse on nonactive synapses.

One important factor that can affect this electrical spillover between synapses is the distance of the nonactivated synapse from the activated synapse. Because voltage attenuates with electrotonic distance in dendrites (Rall, 1967; Rall and Rinzel, 1973), we would naively expect that nonactivated synapses that are closer to the activated synapse will see more dendritic depolarization, and are thus likely to have a larger calcium influx through VGCCs than synapses that are further away from the activated synapse.

### Asymmetric attenuation of EPSPs and NMDA dendritic spikes in dendrites

The distance-dependent attenuation description of heterosynaptic plasticity is complicated by the fact that voltage attenuation in the dendrite is highly asymmetric. For distal dendritic inputs, the proximal dendrites and soma act as a current “sink.” This gives rise to a strong asymmetry of voltage attenuation in dendrites (Rall and Rinzel, 1973).

To demonstrate the effects of dendritic location on voltage attenuation, we created a ball-and-stick cable model with a 200-μm-long cylindrical cable coupled to an isopotential soma (Fig. 2*A*). We enlarged the diameter of the soma to replicate the electrical sink effect that would occur in a layer 5 cortical pyramidal neuron with a full dendritic morphology (Hay et al., 2011; see Materials and Methods; Extended Data Fig. 2-1). We placed a cluster of five dendritic spines with excitatory synapses at 100 μm from the soma. We also placed nonactivated spines at 60 and 200 μm from the soma. We simultaneously activated all synapses in the spine cluster located at 100 μm from the soma and recorded the local voltage at the soma, the dendrite, and at the heads of both the activated and nonactivated spines. Voltage attenuated slightly from the heads of the activated spines to the spine base, but almost no attenuation was visible from the base of the activated spines at 100 μm to the head of the distal nonactive spine at 200 μm. By contrast, the voltage attenuated substantially from the base of the activated spines to the base of the proximal nonactive spine (Fig. 2*B*; Segev and Rall, 1988). This attenuation profile is also qualitatively maintained when the same experiment is performed in a branch from the original pyramidal neuron model or in a ball-and-stick model with uniform *I*_h_ conductance along the dendrite. If *I*_h_ conductances are scaled exponentially along the dendrite, more attenuation may be observed in the distal direction than in the passive cable case (Extended Data Fig. 2-2). Qualitatively, substantial voltage attenuation from spine to dendrite but not from dendrite to spine, is consistent with recent experimental work (Cornejo et al., 2021).

We replicated this experiment with a cluster of 18 synapses at 100 μm from the soma, which was sufficient to generate an NMDA spike in these spines (Eyal et al., 2018). [We note that it is possible to create an NMDA spike with fewer clustered synapses if the synapses are activated at a high frequency (Polsky et al., 2009; Dembrow and Spain, 2022); however, in this work, for simplicity we only vary the cluster size.] The same asymmetric effect as described above was qualitatively observed for the NMDA spike; voltage attenuation was very minor from the activation site to the distal tip, and very substantial from the activation site toward the soma (Fig. 2*B*,*C*).

We next demonstrated how the asymmetric attenuation manifests in a branching dendrite model. We created an order-3 branching dendritic tree (see Materials and Methods) and we simultaneously activated a cluster of 18 synapses in the center of a second-order branch, generating a local NMDA spike there. The NMDA spike propagated to the distal daughter branches with minimal attenuation, propagated to the sister branches of the activated branch and their daughter branches with mild attenuation, and propagated to the rest of the dendritic tree with substantial attenuation (Fig. 2*D*). This same attenuation profile is qualitatively observed if the 18 synapses are not clustered at the exact same location but instead are uniformly dispersed along the 50 μm branch section (Extended Data Fig. 2-3) or if the branching structure of the dendritic tree is asymmetric (Extended Data Fig. 2-4). The stark contrast in the depolarization magnitude of different regions of the dendritic tree in response to a local NMDA spike raises the possibility that asymmetric voltage attenuation may play a functional role in governing plasticity processes in different parts of the dendritic tree.

### Asymmetric voltage attenuation produces asymmetric heterosynaptic plasticity

To explore how asymmetric voltage attenuation can impact heterosynaptic plasticity, we placed a spine at each segment of the ball-and-stick model shown in Figure 3*A1* (one spine every 10 μm) and activated a cluster of 27 synapses at the center of the dendrite. This produced a large NMDA spike at the activated spines, depolarizing the dendrite sufficiently to open VGCCs at nonactivated spines (Fig. 3*A4*).

At the active site, both NMDA channels and VGCCs provided a substantial amount of calcium current into the cell, allowing [Ca^2+^] to surpass $\theta_{P}$, generating homosynaptic potentiation. The depolarization spreading from the activated spines was sufficient to open VGCCs at distal spines, providing enough calcium current to induce heterosynaptic potentiation. However, at a spine located 10 μm proximal to the input site, the voltage had already attenuated sufficiently such that the [Ca^2+^] there following the opening of the local VGCCs induced depression (Fig. 3*A1*,*A5*). At 20 μm proximal to the input site, the voltage attenuated such that the [Ca^2+^] from the VGCCs was insufficient to cross $\theta_{D}$, so the synapses on this spine and all other proximal spines were left unchanged (Fig. 3*A*).

Of course, the specific plasticity outcomes that we observed hold true only for the specific calcium thresholds for plasticity used in our simulation. We therefore performed the same experiment with a variety of different values for the plasticity thresholds. We observe that it is generally easier to induce heterosynaptic plasticity at spines that are distal to the activation site rather than proximal to it (Fig. 3*B*).

This directional asymmetry in heterosynaptic effects is especially pronounced when considering a branching dendrite, as asymmetric attenuation from an input site can create branch-dependent dendritic depolarization (Rall and Rinzel, 1973). To demonstrate this effect, we placed spines every 10 μm on the order-3 branched model described above. We activated clusters of 20, 30, 40, or 50 synapses at the each branching level and observed the plastic changes at all modeled spines (Fig. 4).

10.1523/ENEURO.0014-23.2023.f6-1 Figure 6-1 Vertical heterosynaptic sandwiching in a branched model. ***A1***, Experiment schematic. Clusters of spines are activated at proximal (2nd layer) and distal (4th layer) branches to explore the effect on nonactivated spine on a central branch (3rd layer). ***A2***, Spatiotemporal voltage profiles at the distal (top row), central (middle row), and proximal (bottom row) branches in the cases where a cluster of 40 active synapses are all placed at the center of the proximal branch (left) or distal branch (right), or where two clusters of 20 synapses each are placed on the proximal and distal branches, respectively. ***A3***, Voltage traces at an exemplar spine head from the distal cluster (top), the inactive synapse on the central branch (center), or the proximal cluster (bottom) for each of the experimental protocols (40 proximal, 40 distal, 20 proximal + 20 distal). ***B***, Peak calcium at an exemplar spine on the proximal (left), central (middle), or distal (right) branches, as in Figure 5*C*. ***C***, Plastic effect on each spine as a function of cluster sizes (red, potentiation; blue, depression; white, no change). ***B***, ***C***, same as Figure 6, *B* and *C*, in the main text. Download Figure 6-1, TIF file.

10.1523/ENEURO.0014-23.2023.f6-2 Figure 6-2 Horizontal heterosynaptic sandwiching in a branched model. ***A1***, Experiment schematic. Clusters of spines are activated at the third branching layer on the left and right branches to explore the effect on nonactivated spine on a proximal parent branch (2nd layer). ***A2***, Spatiotemporal voltage profiles at the right (top row), proximal (middle row), and left (bottom row) branches in the cases where a cluster of 40 active synapses are all placed at the center of the left branch (left), right branch (right), or where two clusters of 20 synapses each are placed on the left and right branches, respectively (center). ***A3***, Voltage traces at an exemplar spine head from the right cluster (top), proximal cluster (left), or the inactive synapse on the proximal parent branch (center) for each of the experimental protocols (40 proximal, 40 distal, 20 proximal + 20 distal). ***B***, Peak calcium at an exemplar spine on the left, proximal parent, or right branches, as in Figure 6*B*. ***C***, Plastic effect on each spine as a function of cluster sizes (red, potentiation; blue, depression; white, no change). (Note that to demonstrate plastic effects, calcium thresholds for plasticity used in this figure were different than in other figures; see Materials and Methods.) ***B***, ***C***, same as Figure 6, *E* and *F*, in the main text. Download Figure 6-2, TIF file.

If the activated synaptic cluster is placed on the zero-order branch emerging from the soma, because of the relatively low input resistance there, it is difficult to induce an NMDA spike or homosynaptic plasticity (30 spines are required for depression, 50 are required for potentiation). Even 50 synapses are insufficient in this model to produce an NMDA spike at the most proximal branch, and thus heterosynaptic plasticity is not induced (Fig. 4*A1*). If we move the cluster up to a first-order branch, homosynaptic potentiation is induced with 20 synapses, and an NMDA spike is elicited with 30 synapses. When 40 spines are activated, spines on the dendritic tree distal to the input site are depressed, and when 50 spines are activated, these distal synapses are potentiated (Fig. 4*A2*). When placed at a second-order branch, 20 activated synapses are already sufficient to produce an NMDA spike and heterosynaptic depression at dendritic spines distal to the input site, and 30 spines turns the heterosynaptic depression to potentiation (Fig. 4*A3*). At the third-order branch, the input resistance is sufficiently large to give rise to both homosynaptic and heterosynaptic potentiation at spines distal to the input site with 20 synapses, and with 30 synapses the voltage propagates sufficiently in the proximal direction to depress the sister branches of the input site. The fact that more input was necessary to produce an NMDA spike at proximal locations than distal locations is consistent with experimental findings that distal dendritic locations tend to integrate their input more nonlinearly than proximal locations (Branco and Häusser, 2011). The tiered nature of heterosynaptic plasticity in dendrites, where proximal inputs can induce heterosynaptic plasticity at branches that are distal to it, suggests that dendritic branches might supervise each other in a hierarchical manner. Branches that are closer to the soma (although not so close that it is difficult to generate an NMDA spike) can “teach” the branches that are distal to it because the NMDA spike preferentially propagates backward toward distal locations, leading to heterosynaptic potentiation or depression in descendant branches. Moreover, distal branches with high input resistances may be able to supervise plasticity in their sibling branches via a competitive process where an input sufficient to depress the branch with homosynaptic input will heterosynaptically depress synapses on a sibling branch.

### Synergistic synaptic “sandwiching”

Until now, we have only looked at heterosynaptic effects produced by the activation of a single cluster of colocalized spines, generating a single local NMDA spike. It is possible that multiple clusters can be activated simultaneously, generating diverse depolarization effects in the dendritic tree (Palmer et al., 2014). From a plasticity standpoint, it is important to think about how the clusters of activated synapses can affect each other (through both VGCC-dependent and NMDA-dependent activations) as well as how they affect inactive synapses via heterosynaptic plasticity (through VGCC activation). While it is not feasible to explore the full combinatorial space of cluster activations, we consider a canonical case in our ball-and-stick model where an inactive spine, placed 130 μm from the soma, is “sandwiched” in between two spine clusters, one proximal (60 μm from the soma) and one distal (200 μm from the soma; Fig. 5*A*). This case is important for understanding the mechanisms governing heterosynaptic plasticity because it illustrates the trade-off between two principles. On the one hand, voltage attenuates more steeply toward the soma. On the other hand, it is easier to generate a large/prolonged NMDA spike at distal synapses, because of the higher input resistance at distal locations (Poleg-Polsky, 2015; Doron et al., 2017).

To illustrate this trade-off, suppose we have 40 active synapses to distribute between the proximal cluster and the distal cluster with the goal of maximizing the depolarization, and thus the heterosynaptic calcium influx, at the centrally located inactive synapse. If the input resistance effect dominates, it would be better to place all synapses distally. If the asymmetric voltage attenuation effect dominates, we might assign all 40 synapses to the proximal cluster. In fact, however, it seems that the answer lies in between these two extremes: placing 20 synapses each at the proximal and distal locations results in a slightly larger depolarization at the heterosynaptic synapse than placing all 40 synapses together in a single cluster at either the proximal or distal location (Fig. 5*A*,*B*). The synergy between distal and proximal clusters is not restricted to the case of 40 synapses; for any given number of synapses there appears to be a “sweet spot” for distributing those synapses between the proximal and distal locations to maximize heterosynaptic effects at the central location, albeit with a tendency to assign more synapses to the proximal location (Fig. 5*C*,*D*).

The increased depolarization when the synapses are divided into separate clusters can be explained by the fact that there are diminishing returns for placing additional synapses at the same location because of the reduced driving force when the dendrite is depolarized to near its reversal potential. It is thus better to separate the synapses into separate clusters at locations that are somewhat electrically separated to avoid “wasting” synapses on a dendritic segment that is already maximally depolarized. (Additional synapses can still increase the duration of an NMDA spike when the branch is depolarized to near its electrical reversal; however, for the purposes of heterosynaptic plasticity, it is often crucial to maximize the peak voltage at the heterosynaptic synapse to ensure that the peak calcium through the VGCCs passes the plasticity thresholds). The duration above the plasticity thresholds also affects the magnitude of the plastic changes in the early phase of long-term plasticity, but in the bistable Graupner–Brunel model, magnitude information is lost after several hours in the late phase of plasticity when the synaptic weights are stabilized into a binary UP/DOWN state (Graupner and Brunel, 2012).

The benefit of dividing synapses into two groups is not observed at the proximal and distal locations themselves. While active proximal synapses do increase calcium influx at distal synapses and vice versa (because of both NMDA and VGCC voltage dependence), to maximize peak calcium influx at proximal spines it is best to put all the synapses proximally, and to maximize peak calcium influx at distal spines it is best to put all the synapses distally (Fig. 5*C*,*D*).

The synergistic heterosynaptic sandwiching effect also pertains in a branched neuron model. We placed varying numbers of activated spines on a proximal (first-order) branch and a distal (third-order) branch in our four-layer branched model to observe the heterosynaptic effects at a nonactivated spine on the central (second-order) branch. As in the ball-and-stick model, the peak calcium at the nonactive, central spine was maximized when active synapses were distributed between the proximal and distal branch (Fig. 6*A–C*, Extended Data Fig. 6-1).

In additional to this “vertical sandwiching” scenario, we also explore a “horizontal sandwiching” case, where an inactive spine is placed in the middle of a branch at the second branching layer, and varying numbers of active synapses are placed at its left and right daughter branches at the third branching layer. We again observe in this context that, from the perspective of the inactive spine on the proximal parent branch, dividing the active spines between the left and right daughter branches tends to maximize the peak calcium available for producing heterosynaptic plasticity. As we would expect from the symmetry of the left and right branches relative to the parent branch, the peak heterosynaptic calcium tends to be maximal when the left and right branches have the same number of activated spines (Fig. 6*D–F*, Extended Data Fig. 6-2).

We have thus shown that when an inactive synapse is placed between two synaptic clusters, whether it is vertically sandwiched between a distal and proximal branch or horizontally sandwiched between two of its daughter branches, plasticity-inducing calcium influx tends to be greater than if all the active synapses were placed in a single cluster. This raises the possibility that in addition to the hierarchical supervision effect we showed above, it may be possible to engineer synapse placement in a sophisticated manner to maximize heterosynaptic plasticity induction without requiring an excessive number of active synapses at the same location.

## Discussion

Our simulations have shown a wide range of consequences for synaptic plasticity arising from the hypothesis that heterosynaptic plasticity might result from dendritic depolarization-induced calcium influx through VGCCs. Simple dendritic cable models, combined with model synapses containing NMDA and VGCCs channels, were sufficient to produce spatially sensitive heterosynaptic plasticity effects using a standard calcium-based plasticity mechanism. Specifically, we have demonstrated that a strong dendritic input that generates an NMDA spike can induce heterosynaptic plasticity at dendritic sites that are distal to the input because of asymmetric voltage attenuation in dendrites. This asymmetry can create a hierarchical heterosynaptic effect in a branching dendrite structure, whereby clustered inputs to a branch closer to the soma can act as “supervisors” to synapses located on more distal branches. Moreover, when two input clusters are active, each cluster can increase the plasticity-inducing calcium influx at spines in the other cluster, as well as at nonactive spines. Additionally, calcium influx to a nonactive spine can be maximized by dividing activated spines into two clusters, rather than placing all activated spines at the same location.

The extent to which these phenomena occur in biology remains an open question, and we encourage experimentalists to use the predictions of our model to design experiments to test whether hierarchical heterosynaptic plastic effects indeed occur in the brain. If our predictions are borne out by experiments, then heterosynaptic plasticity can produce a richer repertoire of plastic effects than have been previously considered. If dendritic NMDA spikes indeed act as heterosynaptic supervisors for other (more distal) synapses, the dendritic branching structure and the location of NMDA spike induction become essential for plasticity induction. Location-sensitive NMDA spike-dependent plasticity rules are particularly critical in light of findings that backpropagating somatic action potentials may not reach distal synapses, and therefore do not induce plasticity in these synapses, whereas NMDA spikes can induce plasticity there (Kumar et al., 2021; see also Lisman and Spruston, 2005; Hardie and Spruston, 2009).

Further work can explore diverse neuronal types with different dendritic morphologies to examine whether the branching structure of different neurons may lend themselves to different kinds of plasticity computations. For example, the elaborate fractal branching structure of Purkinje neurons may lend those neurons to be optimized for segregated hierarchical units (Piochon et al., 2007, 2010. (See (Liu et al., 2016) regarding the presence of NMDARs in Purkinje neurons and their influence on plasticity). Conversely, neurons with sufficiently long, branching dendrites (e.g., apical dendrites of L2/3 cells) may exhibit more attenuation in the proximal-to-distal direction and thus behave less hierarchically, as sufficiently long distal branches themselves can act as electrical sinks relative to the parent branch (Landau et al., 2022).

The branch-dependent variation of heterosynaptic plasticity we show in our model is in line with the theory that the dendritic branch may be a fundamental computational unit in the neuron (Koch et al., 1983; Segev and Rall, 1998; Branco and Häusser, 2010). Consistent with the idea that neurons can behave as a two-layer and even a multiple-layer neural network (Poirazi and Mel, 2001; Poirazi et al., 2003; Beniaguev et al., 2021), hierarchical plasticity can potentially serve as a biophysical basis for a multilayer learning algorithm within a single neuron, perhaps akin to the backpropagation algorithm in deep neural networks in feedforward artificial neural networks (Rumelhart et al., 1986; Jones and Kording, 2021). The details of how such an algorithm would operate remain an open avenue for investigation.

One way to make use of these hierarchical and branch-level plastic effects is to take advantage of extant structural input specificity. For example, if a multimodal integration neuron receives auditory input on one branch and visual input on a different branch, a single supervisory NMDA spike can induce the potentiation of all auditory inputs simultaneously, which could be useful in any situation where auditory inputs should be weighted preferentially (e.g., in a low-light environment). If the more distal branching levels also contain additional levels of input specificity (e.g., the visually sensitive dendritic branch might bifurcate into branches sensitive to Gabor-like filters of different frequencies or orientations), a supervisory signal sent to one of these branches might be able to similarly treat one category of inputs preferentially. It should be noted, however, that the assumption that different kinds of sensory inputs make synapses on different dendritic branches is speculative, and some evidence points to a more random, “salt-and-pepper” distribution (Jia et al., 2010).

An alternative possibility is that instead of assuming that each branch receives different categories of inputs, dendritic branches may be sensitive to input features that are correlated in the real world (e.g., the sound of crying with the image of an infant). The hypothesis that correlated inputs are mapped to nearby locations on the dendrite is known as synaptic clustering and has been explored in various theoretical and experimental studies (Mel, 1991, 1992; Kleindienst et al., 2011; Kastellakis et al., 2015; Moldwin et al., 2021).The branch-level plasticity we describe here can operate on these clustered input features to treat the set of clustered inputs at the branch level as a single unit for the purposes of plasticity.

The hierarchical plasticity phenomenon as suggested here is complicated somewhat by our sandwiching results, which demonstrate that, for a given number of activated synapses, heterosynaptic effects can be maximized by distributing them into two (or possibly more) spatially segregated clusters instead of placing them all at the same location. This points to the possibility of an even more sophisticated supervision scheme, where multiple synaptic clusters can be strategically placed at different dendritic locations to produce spatially targeted heterosynaptic plasticity. Spatiotemporally targeted inhibition may also help shape the spread of heterosynaptic plasticity. Further experimental and theoretical work could explore these possibilities in more detail. In any event, the diverse heterosynaptic effects we have shown here provide support for the claim that neurons may behave as complex nonlinear units (Koch and Segev, 2000; Poirazi et al., 2003; Beniaguev et al., 2021; Jones and Kording, 2021; Larkum, 2022) as opposed to simple perceptrons where synapses are modified independently (Moldwin and Segev, 2020). Moreover, the pronounced asymmetrical voltage attenuation in dendrites and the attendant consequences for heterosynaptic plasticity shown in our simulations indicate that computational models that make use of distance-dependent NMDA superlinearities (Mel, 1991; Moldwin et al., 2021) should take into account branching structure and synaptic location relative to the soma in addition to the relative distance of synapses from each other.

Our model, in line with the proposal of Lisman (2001), assumes that the only medium of communication between active and inactive synapses is dendritic voltage depolarization, which can activate VGCCs of other nonactivated dendritic spines. We note that many other mechanisms for the induction of heterosynaptic plasticity have been suggested (for review, see Chistiakova et al., 2014; Chater and Goda, 2021). One alternative possibility is that calcium itself diffuses from one synapse to another; however, experimental evidence suggests that calcium diffusion from the spine head into the dendritic shaft is negligible (Yuste and Denk, 1995; Sabatini et al., 2002). Other molecules have also been implicated in inducing heterosynaptic effects, such as h-Ras, Rac1, RhoA, Arc, BDNF-TrkB, CaMKII, and calcineurin (Chater and Goda, 2021; Tong et al., 2021); however, these molecules have only been shown to diffuse up to 10 μm along the dendrite, while heterosynaptic effects have been shown to occur at much larger distances between activated and nonactivated synapses (Lynch et al., 1977; Engert and Bonhoeffer, 1997). As such, the depolarization-based model remains an important candidate mechanism of heterosynaptic plasticity. It may be that there are different short-distance and long-distance heterosynaptic effects, with short-distance effects occurring via molecular mechanisms such as local CamKII and calcineurin activity, while long-distance effects may be because of the voltage mechanism we describe here.

Regarding the fidelity of the parameters in our simulation to biological reality, there are several questions that would require additional experimental evidence and more detailed models to fully confirm. Our calcium channel model assumed a single type of calcium channel, and we chose a conductance value that approximately corresponds to what we might expect as the aggregate conductance of all high-voltage activated VGCCs. A more precise model that includes all forms of VGCCs with their appropriate unitary conductances, kinetics, and densities would allow us to examine our claims with greater precision. Additionally, the kinetics of calcium accumulation and the plasticity thresholds for calcium used here could be better constrained with more experimental evidence. We also assumed that calcium channel density and plasticity thresholds were the same from spine to spine; in biology, these may differ on a spine-by-spine basis even with a single neuron. Moreover, the spatial effects we observed in our simulations assume a passive dendritic cable; active mechanisms in biological dendrites such as voltage-gated sodium, calcium, and potassium channels have been shown to differentially modulate voltage propagation in different neurons (Golding et al., 2001), so these mechanisms would consequently be expected to modify the spatial dynamics of heterosynaptic plasticity as well.

Inhibitory synapses also likely play an important role in the spatial reach of heterosynaptic plasticity. Inhibition can have different consequences for the neuronal voltage depending on the location of the inhibitory synapses (Gidon and Segev, 2012; Jadi et al., 2012, 2014; Bar-Ilan et al., 2013) as well as their timing relative to excitatory NMDA inputs (Doron et al., 2017). As such, spatiotemporally targeted inhibition can be used to modulate the heterosynaptic effects we describe here, enabling a bidirectional control system for heterosynaptic plasticity.

Several studies have shown that internal calcium stores play an important role in both homosynaptic and heterosynaptic plasticity (Nishiyama et al., 2000; Rose and Konnerth, 2001; Royer and Paré, 2003; Jo et al., 2008; Camiré and Topolnik, 2014; Evans and Blackwell, 2015; O’Hare et al., 2022). Ryanodine and IP_3_ receptors produce calcium-induced calcium release (CICR), which can affect plasticity in a variety of ways. Although experimental results regarding the role of CICR are more subtle than the model we have presented here, one way to think about CICR is as an amplifier of calcium coming from NMDA and voltage-gated calcium channels. As such, assuming that CICR increases monotonically with calcium from extracellular sources, the basic qualitative principle that activated synapses will experience more calcium release than nonactivated synapses because of NMDA receptor activation still holds, but it may shift the homosynaptic and heterosynaptic effects observed (e.g., from homosynaptic depression and no heterosynaptic effect to homosynaptic potentiation and heterosynaptic depression; Fig. 1*C*). However, there is evidence that the effect of internal calcium stores is highly localized into microdomains and depends on various second messengers, resulting in a more complex picture of homosynaptic and heterosynaptic effects (Evans and Blackwell, 2015).

Another biological mechanism that may affect the plastic results we predict here are small-conductance Ca^2+^-activated K^+^ channels (SK channels). SK channels can repolarize the membrane in response to calcium influx (Adelman et al., 2012; Tigaret et al., 2016; Rodrigues et al., 2021), potentially reducing both homosynaptic and heterosynaptic effects.

One additional crucial biological question is whether the early stage heterosynaptic plasticity induced by calcium influx is stabilized into late-term plasticity via protein synthesis, which has been shown to be necessary to make plastic changes last longer than an hour (Frey and Morris, 1997; Barco et al., 2008; Redondo et al., 2010). One recent study (Sun et al., 2021) showed that plasticity-induced protein synthesis may primarily occur within 3 μm of potentiated synapses, suggesting that heterosynaptic effects may not necessarily be long lived. However, it is possible that a very strong clustered stimulation, such as we described here, may induce protein synthesis at more distant locations.

## Synthesis

Reviewing Editor: Arvind Kumar, KTH Royal Institute of Technology Decisions are customarily a result of the Reviewing Editor and the peer reviewers coming together and discussing their recommendations until a consensus is reached. When revisions are invited, a fact-based synthesis statement explaining their decision and outlining what is needed to prepare a revision will be listed below. The following reviewer(s) agreed to reveal their identity: Sahil Moza.

Your manuscript was reviewed by two reviewers. Both have in general appreciated the work and agreed that the work is an important contribution to our understanding of calcium-based synaptic plasticity and role of neuron morphology in shaping the synaptic plasticity. The manuscript is very well written and the results are clear. But the two reviewers have also raised a few issues related to the validity of results in a more biologically plausible settings e.g.

- How will the results change when we consider that active conductance can compensate for the voltage attenuation along the dendrite

- How will the results change if you were to consider an asymmetric dendritic arbor

- Do the results depend on the spatial distribution of the spines

- Will ongoing activity related fluctuations in the membrane potential obscure the observed heterosynaptic plasticity

We understand that all this may not be feasible to do in a short time but we expect that you include some examples simulations showing to what extent the aforementioned points may affect the results.

We look forward to receiving the revised version of the manuscript. In the following the detailed comments from the reviewers are appended. You should revise the manuscript accordingly.

Reviewer #1

In this paper, the authors have analyzed the repertoire of synaptic plasticity resulting from the interaction between the asymmetry due to the hierarchical dendritic structure, voltage-dependent and ligand-dependent (NMDA) or independent (VGCCs) mechanisms of calcium influx into dendritic spines, and a calcium-based variation of the BCM-rule. The central argument of the paper is that there is a rich space of plasticity outcomes resulting from the differences in calcium concentration in spines that i) get direct glutamatergic input and ii) those that only receive calcium influx from voltage-gated calcium channels (VGCC) activated by membrane depolarization due to propagating NMDA spikes from neighboring regions. For example, the spines getting only VGCC can get depressed, because they are above the “no effect” threshold but below the potentiation threshold. Since there is an asymmetry in the electrotonic properties of neurons from basal to apical regions, the hierarchical position of spines on the dendritic arbor and the amount of clustered and synchronous activation can affect which spine or even all spines on a dendritic branch get potentiated or depressed. Overall, this is a valuable study for computational neuroscientists interested in synaptic plasticity and electrophysiologists who might be able to test heterosynaptic plasticity induction by selecting input intensity and position based on their analysis. However, the study has a few key assumptions and conceptual problems that need to be substantiated or addressed.

1. Active channel gradients can affect the electrotonic voltage drop:

There are compensatory mechanisms that mitigate the electrotonic asymmetry in the distal dendrites, such as based on Ih channel gradients. Since the authors premise their argument on the asymmetry of the dendritic arbor, and the local computations on dendrites, it would be important to note the effect of such active channel mechanisms on their analysis, given that these mechanisms can compensate for electrotonic dendritic filtering.

Minor:

1. The distribution of spines on the dendrites may not be uniform.

It would be good to know how strongly the analysis depends on the idea that the spacing between spines stays identical across the different hierarchical levels of the dendritic arbor. The spine distribution may vary between the basal and apical part of the dendritic arbor, affecting the direct comparison of clustered inputs in these locations.

Discussion:

Line 384, 385: The details of how such an algorithm would operate remain an open avenue for investigation.

It would be good if the authors elaborate a bit on how heterosynaptic plasticity would be a part of a multi-layer learning algorithm, or add to the already existing ideas of neurons as a multi-layered perceptron. This section otherwise lacks a bit of specificity.

The blue color in Fig 5,6 obfuscates the text below, and preferably should be a part of the figure colorbar.

Typos:

Line 422: Calcneurin -> Calcineurin

Line 478 (NERUON) -> NEURON

Citations: The paper has missed a few citations such as Losonczy and Magee, 2002; Branco and Hausser 2011, which provide important reference points to put this paper in context.

Reviewer #2

First off, it’s a very well written, extremely pedagogical and impressive study! Starting with well known facts, authors gradually build and develop their concept making an interesting story. I personally have no objections to the publication but need to clarify a few points.

1) Using a ball-and-stick model and adjusting the soma size is perfectly fine to match the somatic input resistance. In this case, the enlarged soma accounts for all missing dendritic branches. However, for the synaptic inputs you study, the local impedance at the distal locations and the effective size of the sink are important. Unfortunately, this discussion is completely omitted with only brief reference to Supplementary 1A (L494-5) where all analysis is done. I think it would be helpful to provide more explanation of Supplementary Figure S1 in the Methods.

2) Results with branched dendritic model are interesting. Unfortunately, the study is limited to a fully symmetric tree. Since it’s a rare case in reality, it would be good to discuss or show an example with a different asymmetry index.

3) Parameter selection for the branching dendrite model (L195-7) is not explained. Also, not clear how it relates to the ball-and-stick introduced earlier or original L5PC.

4) No results are illustrated using the original L5PC model.

5) Great that the basic assumptions are listed starting at L132. However, not all of them are mentioned in that paragraph as they are discussed later in “Additional biological considerations” in more detail.

6) Interesting results with “sandwiched” synapses! Did authors try a possibility of the sparse synapse placement within a given branch instead of the clustered one?

Minor notes.

L100: extra “be” in “short-range effects can be potentially be explained”.

L144: extra “assumptions” in “assumptions our assumptions above”.

L300: possibly missing “order” in “in [order] to ensure”.

L302-3: possibly extra “bistable” in “in the bistable Graupner-Brunel bistable model”.

L313-9: inconsistent use of “layer” and “order” in the text and the legend of Figure 6. Probably better to use “order” here since the topological order of the dendritic sections within the dendritic tree is meant.

--

Comments about the extended data and software:

Extended Data: Full code for the cell models is provided. No README file explaining how to run the example scripts and what output to expect. The code does not help much as it is not clean. Few scripts contain hard coded absolute paths and don’t run out-of-the-box.

Software Comments: The manuscript refers to the standard tools, Python v3.7.6, NEURON v7.7.2 and a BBP synaptic model. It is not clear which version of the synaptic model is used though. Reference to Chindemi at bioRxiv can now be changed to Nature Communications doi: 10.1038/s41467-022-30214-w, with specified version number of supplementary source code on Zenodo and particular path/file name, e.g. ProbAMPANMDA_EMS.mod.
