# Erratum: Moldwin et al., “Asymmetric Voltage Attenuation in Dendrites Can Enable Hierarchical Heterosynaptic Plasticity”

**DOI:** 10.1523/ENEURO.0032-24.2024

**Published:** 2024-02-09

In the article “Asymmetric Voltage Attenuation in Dendrites Can Enable Hierarchical Heterosynaptic Plasticity,” by Toviah Moldwin, Menachem Kalmenson, and Idan Segev, which was published online on July 6, 2023, the red–white–blue color bars were incorrectly labeled in Figures 5, 6, 6-1, and 6-2. The red portion of the bar should be labeled with “Potentiation” and blue portion of the bar should be labeled “Depression.” This correction does not affect the conclusions of the paper. The article has been corrected online and corrected versions of the figures appear below.

**Figure:**
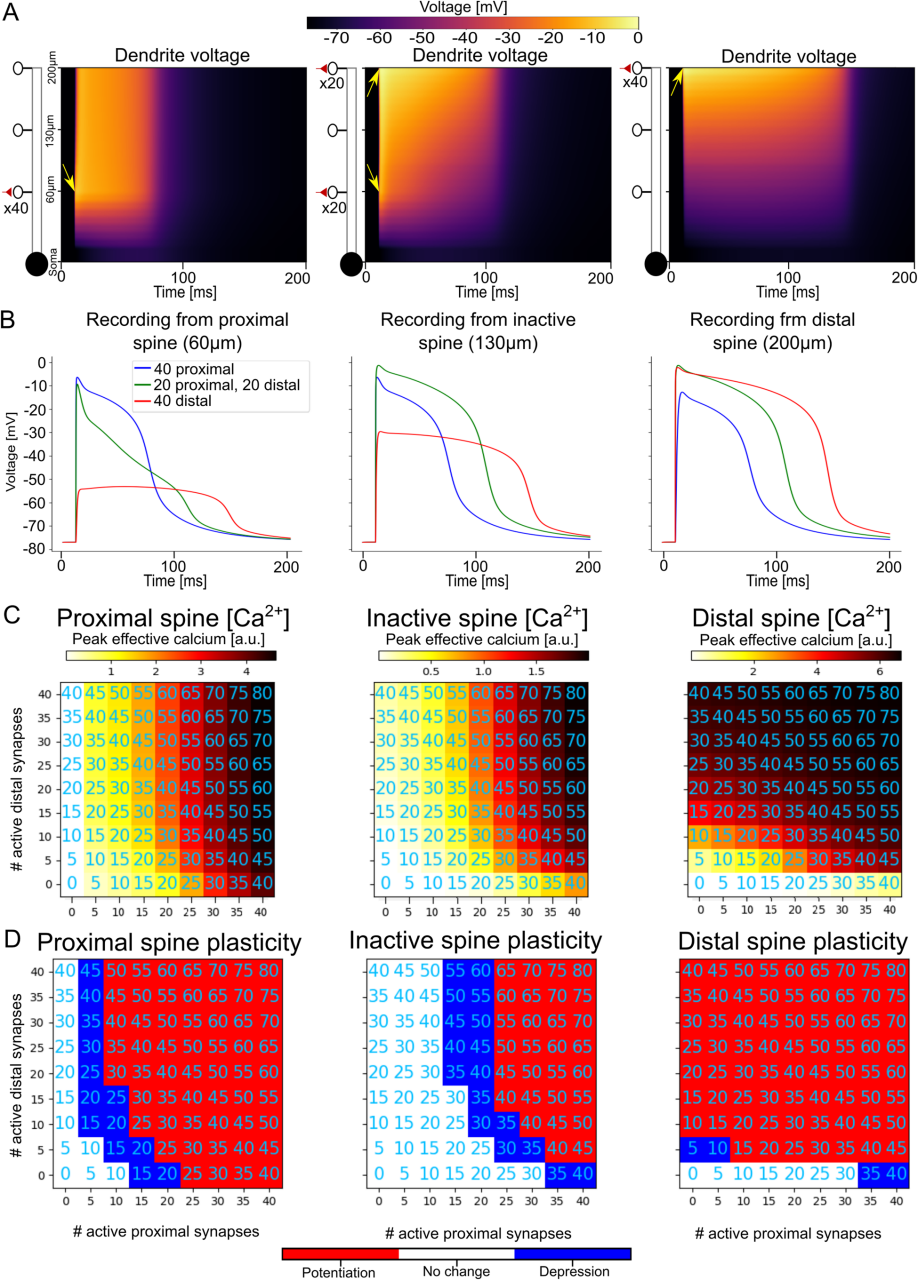
Figure 1.

**Figure:**
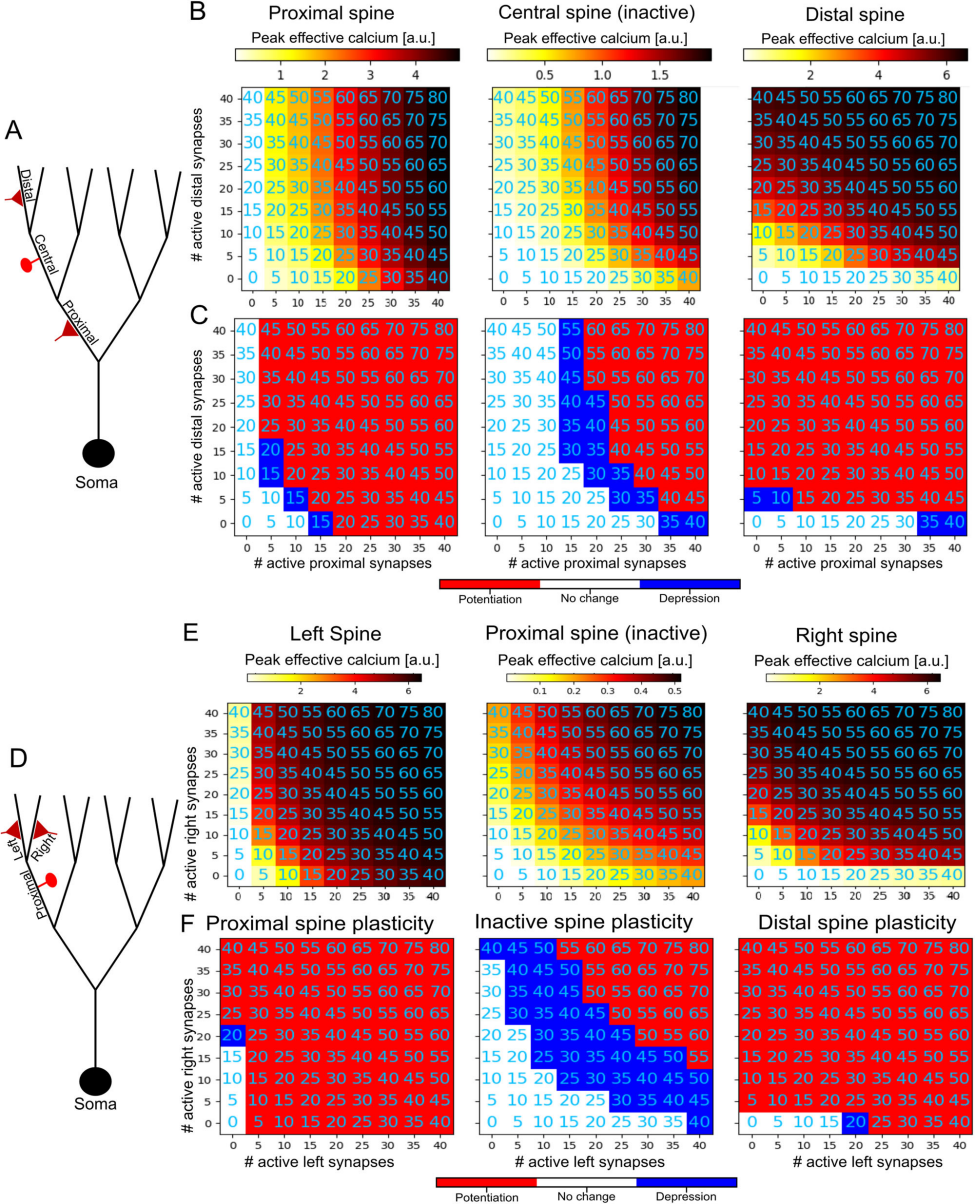
Figure 2.

**Figure:**
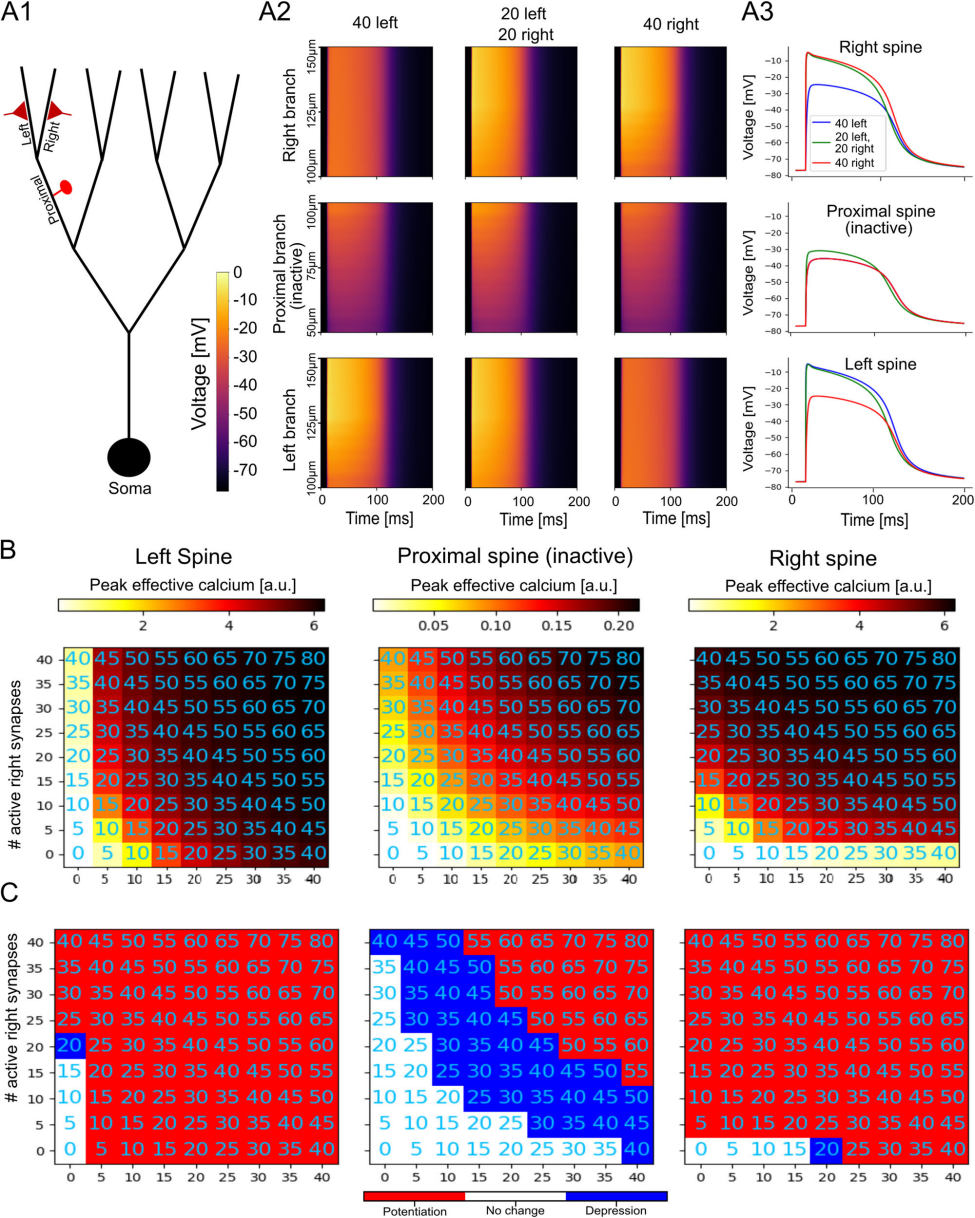
Figure 3.

**Figure:**
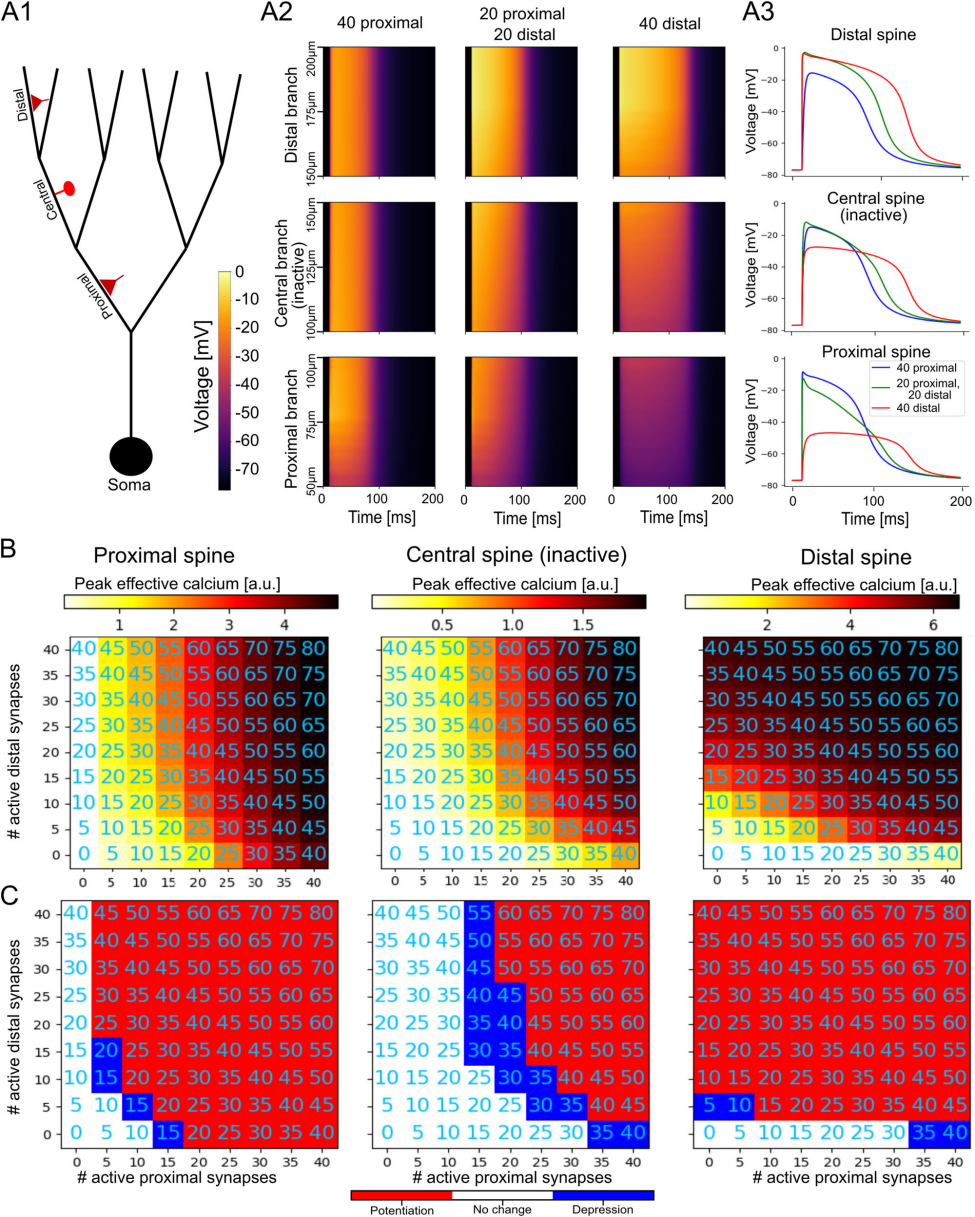
Figure 4.

