# Supplementary figures and images for: Asymmetric Voltage Attenuation in Dendrites Can Enable Hierarchical Heterosynaptic Plasticity
Source: eNeuro. 2023 Jul 14;10(7):ENEURO.0014-23.2023. doi: 10.1523/ENEURO.0014-23.2023 (PMC10354808; doi:10.1523/ENEURO.0014-23.2023)

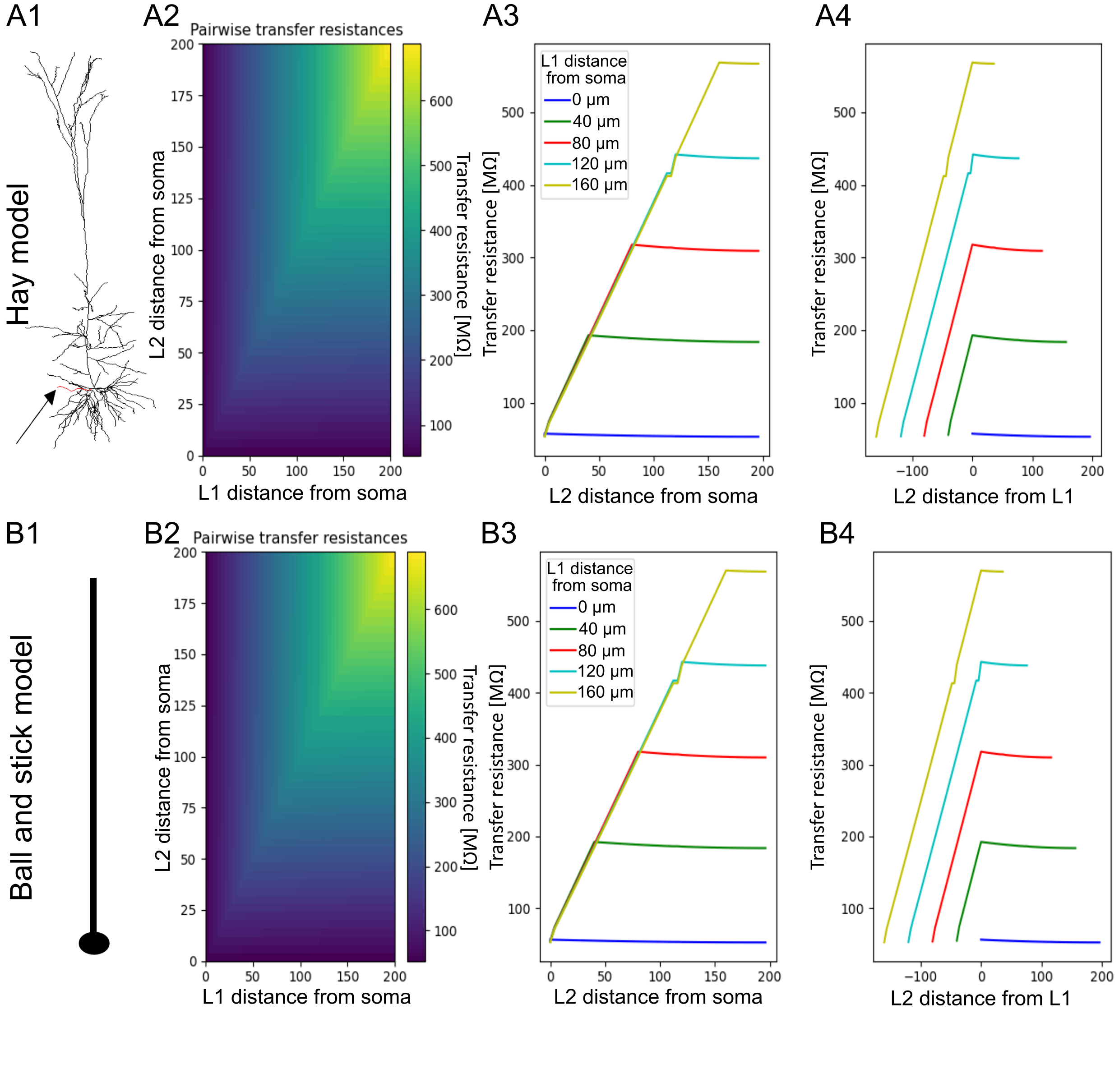

Supplement: Figure 2-1 — Asymmetric transfer resistances of L5PC and ball-and-stick models. A1, Model of layer 5 pyramidal cell from the study by Hay et al. (2011). Arrow indicates a dendrite modified to have the same morphological and passive electrical parameters as the idealized ball-and-stick model. A2, Pairwise transfer resistances between each dendritic segment, presented as heatmap. L1. location 1, L2, location 2. A3, Transfer resistances from selected dendritic locations (L1) to all other dendritic locations (L2) as a function of distance of L2 from the soma. A4, Same as in A3, except L2 is depicted as a function of distance from L1, demonstrating asymmetric attenuation from each location. Negative numbers indicate L2 is more proximal than L1, and positive numbers indicate L1. B1–B4, Same as in A1–A4, but for the ball-and-stick model used in the article with the soma diameter adjusted to match the somatic input resistance (RN) from the Hay L5PC model. Download Figure 2-1, TIF file. [file enu-eN-NWR-0014-23-s01.tif]

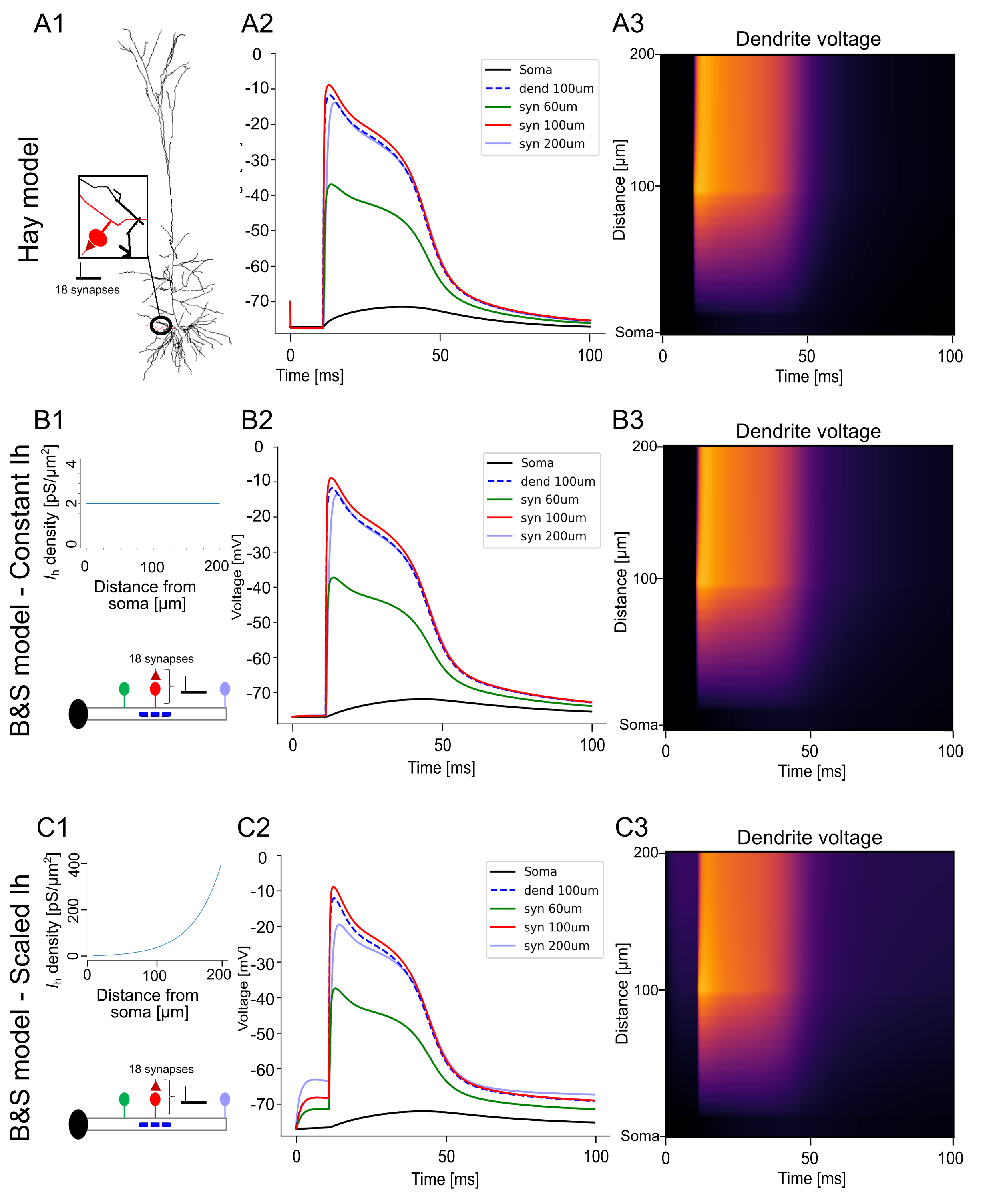

Supplement: Figure 2-2 — Attenuation in dendritic branches with active channels. A1, A cluster of 18 synapses are activated in the center of the modified branch from the study by Hay et al. (2011) model with active channels. A2, Voltage traces from the activation location (100 μm from soma) and other locations on the branch and soma. Compare with Figure 2C2. A3, Heatmap of dendritic activation over space and time in the dendritic branch model. Compare with Figure 2C3. B1, Same experiment performed on ball-and-stick model with a constant (as a function of distance from soma) Ih conductance of 2 pS/μm2 along the dendrite, as in the basal dendrites of the original model from the study by Hay et al. (2011). C, Same experiment performed in ball-and-stick model with Ih conductance scaled with distance from soma such that the Ih conductance at the distal tip is near 400 pS/μm2 to imitate the Ih found in distal apical dendrites of pyramidal neurons 1000 μm from the soma (Kole et al., 2006). Scaling was performed according to the equation gIh=−2+428+ex0.135*323 pS/μm2, where x is the distance from the soma. Download Figure 2-2, TIF file [file enu-eN-NWR-0014-23-s02.tif]

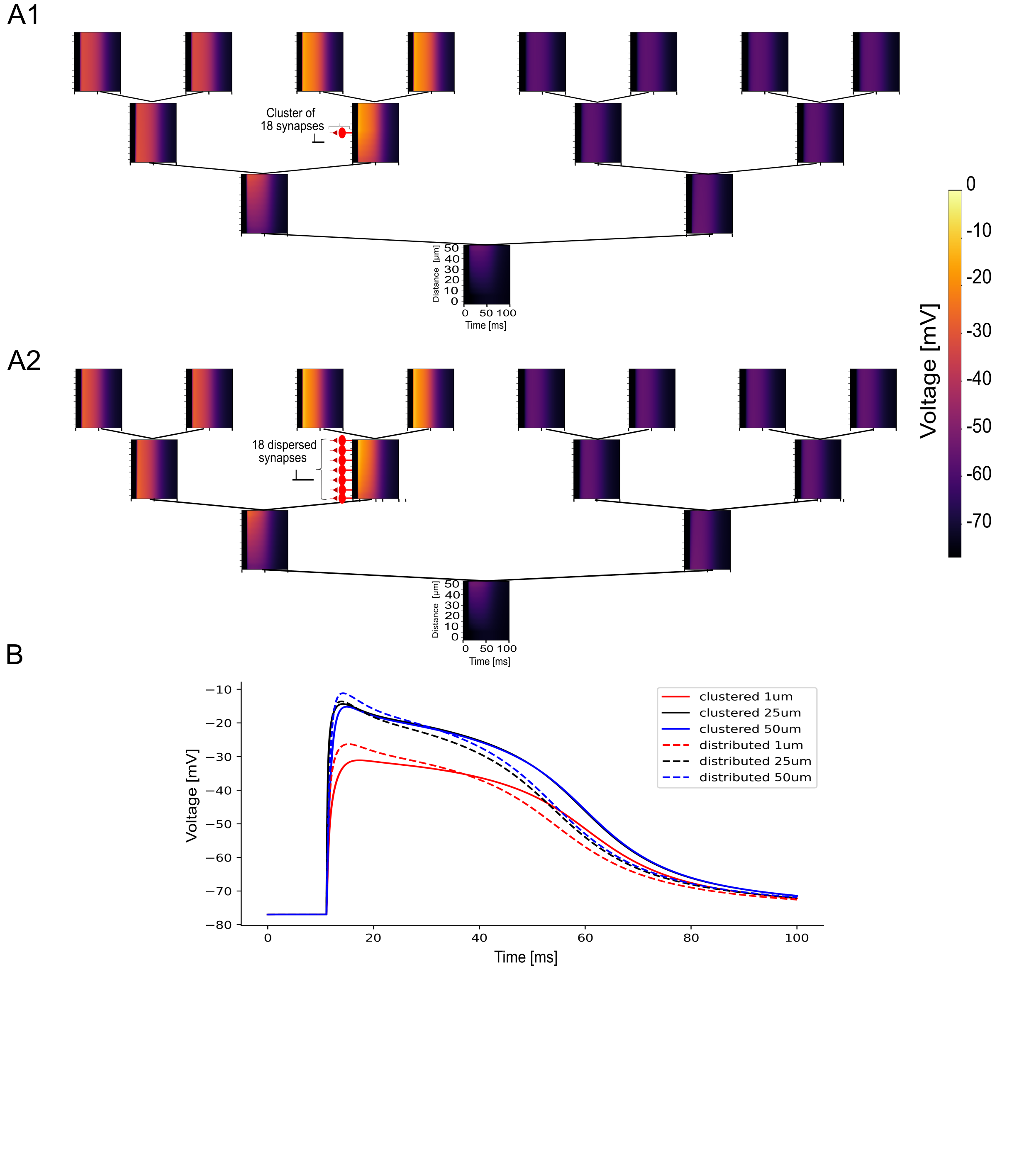

Supplement: Figure 2-3 — Effect of clustered versus distributed synaptic distribution. A1, Attenuation in branching dendritic model with synapses clustered at a single dendritic segment, as in Figure 2D. A2, Same as in A1, except synapses are uniformly distributed along the branch (18 synapses spread over 50 μm, a density of 0.36 synapses/μm). B, Comparison of voltage traces from the proximal and distal ends (1 and 50 μm, respectively) and midpoint (25 μm) of the activated branch in the clustered and distributed scenarios. Download Figure 2-3, TIF file. [file enu-eN-NWR-0014-23-s03.tif]

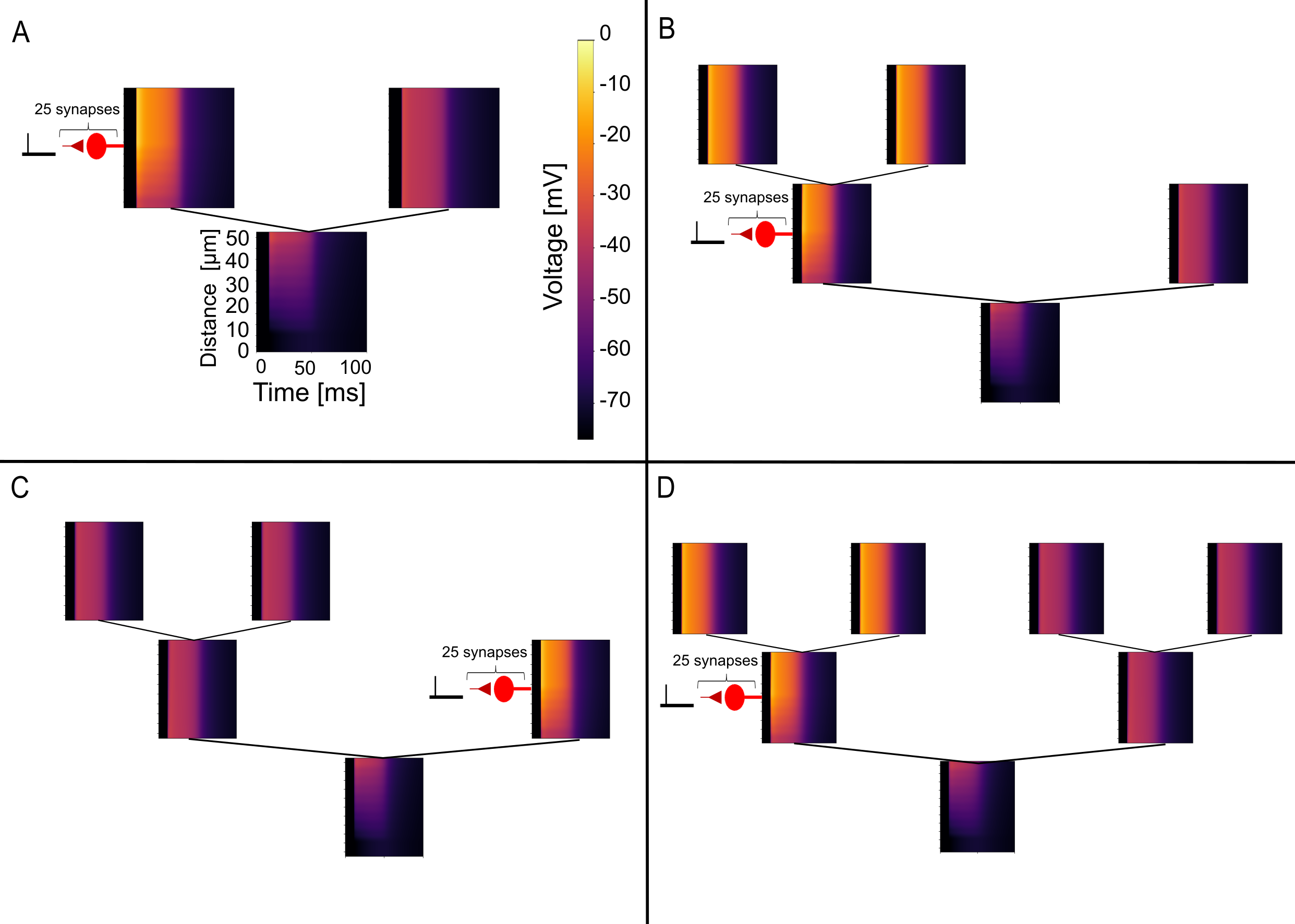

Supplement: Figure 2-4 — Attenuation in asymmetric dendritic morphologies. A, B, Synapses are placed in the indicated location on a dendritic tree with different morphologies: order-1 branching dendrite (A); and asymmetric dendritic morphology with additional branching level on the left side with synapse placed on left branch (B). C, As in B but synapse placed on the right branch. D, Order-2 dendritic tree. Download Figure 2-4, TIF file. [file enu-eN-NWR-0014-23-s04.tif]

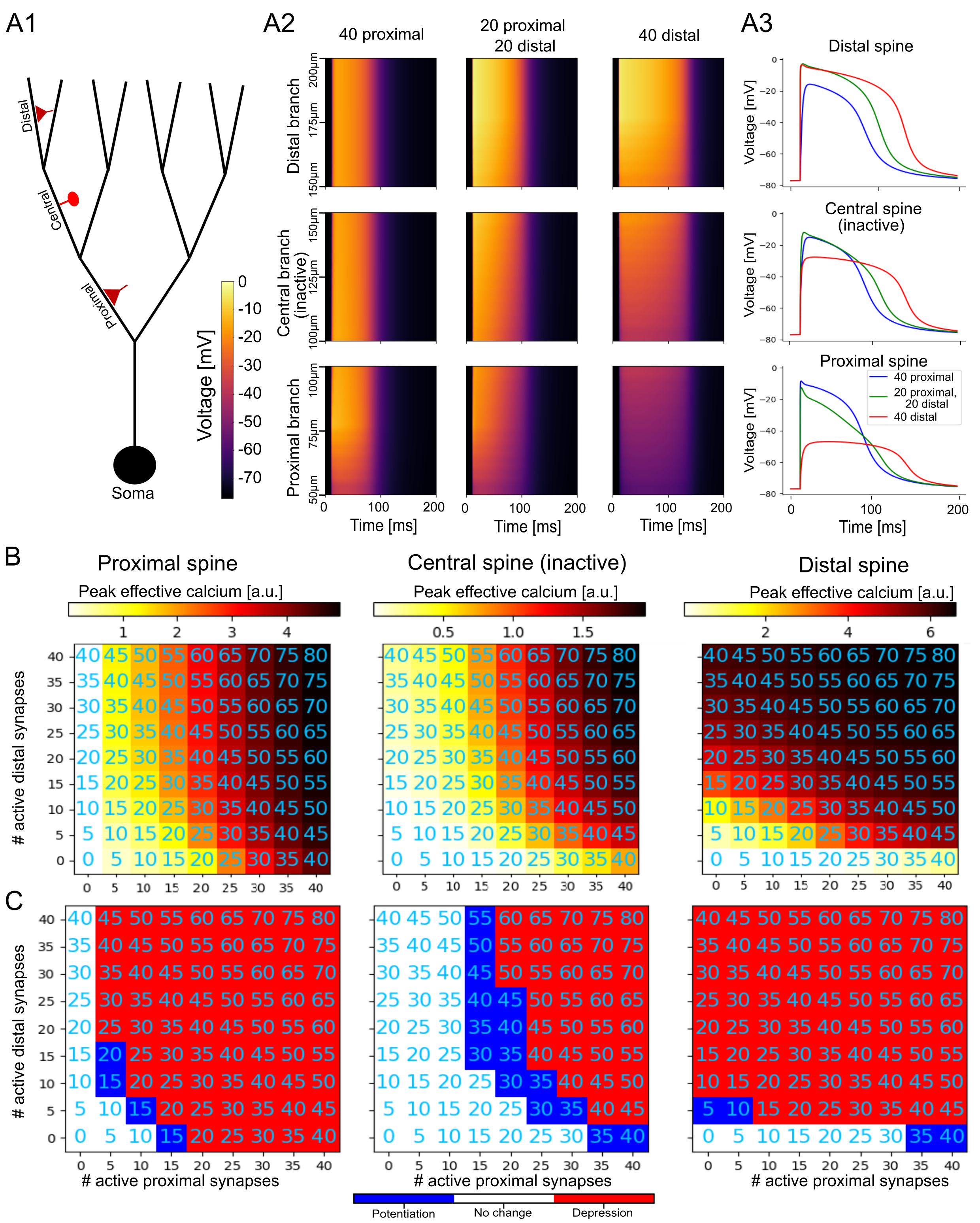

Supplement: Figure 6-1 — Vertical heterosynaptic sandwiching in a branched model. A1, Experiment schematic. Clusters of spines are activated at proximal (2nd layer) and distal (4th layer) branches to explore the effect on nonactivated spine on a central branch (3rd layer). A2, Spatiotemporal voltage profiles at the distal (top row), central (middle row), and proximal (bottom row) branches in the cases where a cluster of 40 active synapses are all placed at the center of the proximal branch (left) or distal branch (right), or where two clusters of 20 synapses each are placed on the proximal and distal branches, respectively. A3, Voltage traces at an exemplar spine head from the distal cluster (top), the inactive synapse on the central branch (center), or the proximal cluster (bottom) for each of the experimental protocols (40 proximal, 40 distal, 20 proximal + 20 distal). B, Peak calcium at an exemplar spine on the proximal (left), central (middle), or distal (right) branches, as in Figure 5C. C, Plastic effect on each spine as a function of cluster sizes (red, potentiation; blue, depression; white, no change). B, C, same as Figure 6, B and C, in the main text. Download Figure 6-1, TIF file. [file enu-eN-NWR-0014-23-s05.tif]

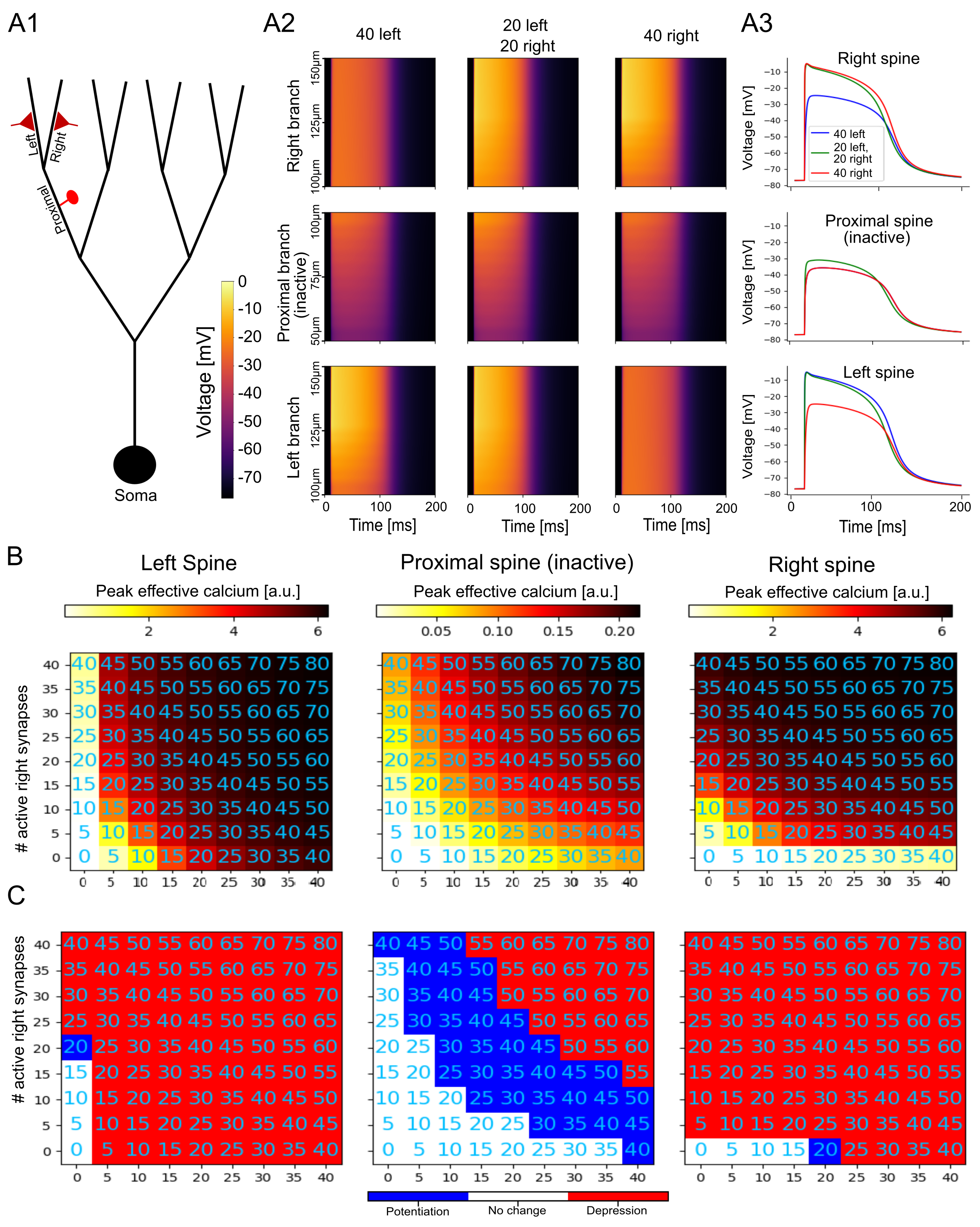

Supplement: Figure 6-2 — Horizontal heterosynaptic sandwiching in a branched model. A1, Experiment schematic. Clusters of spines are activated at the third branching layer on the left and right branches to explore the effect on nonactivated spine on a proximal parent branch (2nd layer). A2, Spatiotemporal voltage profiles at the right (top row), proximal (middle row), and left (bottom row) branches in the cases where a cluster of 40 active synapses are all placed at the center of the left branch (left), right branch (right), or where two clusters of 20 synapses each are placed on the left and right branches, respectively (center). A3, Voltage traces at an exemplar spine head from the right cluster (top), proximal cluster (left), or the inactive synapse on the proximal parent branch (center) for each of the experimental protocols (40 proximal, 40 distal, 20 proximal + 20 distal). B, Peak calcium at an exemplar spine on the left, proximal parent, or right branches, as in Figure 6B. C, Plastic effect on each spine as a function of cluster sizes (red, potentiation; blue, depression; white, no change). (Note that to demonstrate plastic effects, calcium thresholds for plasticity used in this figure were different than in other figures; see Materials and Methods.) B, C, same as Figure 6, E and F, in the main text. Download Figure 6-2, TIF file. [file enu-eN-NWR-0014-23-s06.tif]
